# Ribosomal RNA fragmentation into short RNAs (rRFs) is modulated in a sex- and population of origin-specific manner

**DOI:** 10.1186/s12915-020-0763-0

**Published:** 2020-04-13

**Authors:** Tess Cherlin, Rogan Magee, Yi Jing, Venetia Pliatsika, Phillipe Loher, Isidore Rigoutsos

**Affiliations:** Computational Medicine Center, Jefferson Alumni Hall #M81, Thomas Jefferson University, 1020 Locust Street, Philadelphia, PA 19107 USA

**Keywords:** Ribosomal RNA, rRNA, rRNA-derived fragments, rRFs, microRNA, miRNA, isomiRs, transfer RNA, tRNA-derived fragments, tRFs, 1000 Genomes Project

## Abstract

**Background:**

The advent of next generation sequencing (NGS) has allowed the discovery of short and long non-coding RNAs (ncRNAs) in an unbiased manner using reverse genetics approaches, enabling the discovery of multiple categories of ncRNAs and characterization of the way their expression is regulated. We previously showed that the identities and abundances of microRNA isoforms (isomiRs) and transfer RNA-derived fragments (tRFs) are tightly regulated, and that they depend on a person’s sex and population origin, as well as on tissue type, tissue state, and disease type. Here, we characterize the regulation and distribution of fragments derived from ribosomal RNAs (rRNAs). rRNAs form a group that includes four (5S, 5.8S, 18S, 28S) rRNAs encoded by the human nuclear genome and two (12S, 16S) by the mitochondrial genome. rRNAs constitute the most abundant RNA type in eukaryotic cells.

**Results:**

We analyzed rRNA-derived fragments (rRFs) across 434 transcriptomic datasets obtained from lymphoblastoid cell lines (LCLs) derived from healthy participants of the 1000 Genomes Project. The 434 datasets represent five human populations and both sexes. We examined each of the six rRNAs and their respective rRFs, and did so separately for each population and sex. Our analysis shows that all six rRNAs produce rRFs with unique identities, normalized abundances, and lengths. The rRFs arise from the 5′-end (5′-rRFs), the interior (i-rRFs), and the 3′-end (3′-rRFs) or straddle the 5′ or 3′ terminus of the parental rRNA (x-rRFs). Notably, a large number of rRFs are produced in a population-specific or sex-specific manner. Preliminary evidence suggests that rRF production is also tissue-dependent. Of note, we find that rRF production is not affected by the identity of the processing laboratory or the library preparation kit.

**Conclusions:**

Our findings suggest that rRFs are produced in a regimented manner by currently unknown processes that are influenced by both ubiquitous as well as population-specific and sex-specific factors. The properties of rRFs mirror the previously reported properties of isomiRs and tRFs and have implications for the study of homeostasis and disease.

## Background

rRNAs are the most abundant RNA molecules in eukaryotic cells [1, 2]. Processed rRNAs are modified and bound to ribosomal proteins to help make up the small and large subunits of the ribosomes [3, 4]. In the ribosome, rRNAs form highly conserved secondary structures in order to complex with ribosomal proteins while also recognizing motifs on transfer RNA (tRNA) and messenger RNAs (mRNAs) [5]. Four rRNAs (5S, 5.8S, 18S, and 28S) are encoded by the human nuclear genome whereas two additional ones (12S and 16S) are encoded by the mitochondrial (MT) genome [6, 7].

Each of the four nuclear rRNAs has multiple copies scattered across the nuclear genome [6]. Three of the four rRNAs, namely 18S, 5.8S, and 28S, are transcribed and processed in the nucleolus from a single precursor molecule, the 45S rRNA [6]. A fourth rRNA, 5S, is transcribed independently and later transferred to the nucleolus where it combines with the 5.8S and 28S rRNAs to form the large ribosomal subunit (LSU) [6, 8]. There is also a small ribosomal subunit (SSU) whose rRNA component is the 18S rRNA. Both the SSU and LSU are assembled in the nucleus before they are transported to the cytoplasm [8]. Once in the cytoplasm, the SSU and LSU combine to form functional ribosomes [3].

The two MT rRNAs serve the same functions analogous to those of their nuclear counterparts [7]. They are transcribed from the circular MT genome in a polycistronic fashion and processed in MT foci called nucleoids [7, 9]. Of the two, 16S combines with mito-ribosomal proteins to form the MT LSU. Likewise, 12S rRNA combines with mito-ribosomal proteins to form the MT SSU in the mitochondriolus. Interestingly, the 5S rRNA is also found in the mitochondria and has been shown to be required for the translational function of the mitochondrial ribosome [10].

The 45S rRNA gene clusters or “cassettes” are located in tandem repeats on the p-arms of the acrocentric chromosomes 13, 14, 15, 21, and 22 [3, 11]. Their exact copy numbers vary from person to person but have been estimated to be between ~ 60 and ~ 800 copies per haploid genome [2, 12, 13]. Similarly, the 5S rRNA exists in tandem repeats predominantly on chromosome 1 with 10–400 copies per haploid genome [2, 14]. Because they are vital for the translation machinery of a cell, rRNA genes are hypo-methylated and transcribed rapidly and more frequently than other genes [3].

The MT rRNAs can also be transcribed independently and at a higher rate than the other 22 tRNAs and 13 protein-coding genes that are also encoded by the MT genome. This independent transcription is accomplished by a unique transcription-termination sequence that is located at the boundary between the 16S rRNA and the downstream mitochondrial tRNA^Leu^ [9]. Of note, the number of MT genome copies varies between MTs within a cell, from cell to cell, and from person to person: this has the potential to impact the overall abundance of MT rRNAs [15, 16].

Increasingly, analyses of deep-sequencing datasets have been drawing attention to the presence of short RNAs that are produced routinely and abundantly from all six rRNAs [17, 18]. These fragments, henceforth referred to as “rRNA-derived fragments” or rRFs, have been reported in multiple organisms including human [19]. These emerging findings mirror previous reporting that each miRNA arm produces a “cloud of isomiRs” [20] and that precursor and mature tRNAs produce “clouds of tRFs” as well [21, 22].

The studies of rRFs become particularly relevant when considered in the context of our long-standing work with microRNAs (miRNAs), microRNA isoforms (isomiRs), transfer RNAs (tRNAs), and tRNA-derived fragments (tRFs). In a series of articles, we showed that the clouds of isomiRs are produced *constitutively* in human tissues, in health [23] and disease [24, 25]. Moreover, we showed that a person’s race, population origin, and sex modulate the clouds of isomiRs in health [23] and disease [24] and do so in a tissue-specific manner [25]. In complete analogy to the isomiRs, we also showed that the clouds of tRFs are also produced *constitutively* in human tissues, in health [26, 27] and disease [28–31], and are modulated by a person’s race, sex, and population origin, as well as by tissue type and tissue state, in health and disease [26, 28–31].

In what follows, we examine whether rRFs exhibit properties analogous to those we reported previously for isomiRs and tRFs. We first focus on a public collection of transcriptomic data that are part of the 1000 Genomes Project in order to understand global and population-specific rRF characteristics [32]. The collection comprises short RNA-seq datasets from many healthy individuals, representing both sexes evenly, five population groups, and two continents. Specifically, we investigate the production of rRFs, within and across populations, and separately for each sex, and for each of the six rRNAs. We also examine the presence of rRFs in 80 uveal melanoma samples [33] and 293T cells and extracellular vesicles [34]. Lastly, we evaluate whether the profiles of rRFs change when samples are processed by different laboratories or sequenced using different library preparation kits.

## Results

### Overview of the rRF RNA-seq analysis pipeline

**Fig. 1:**
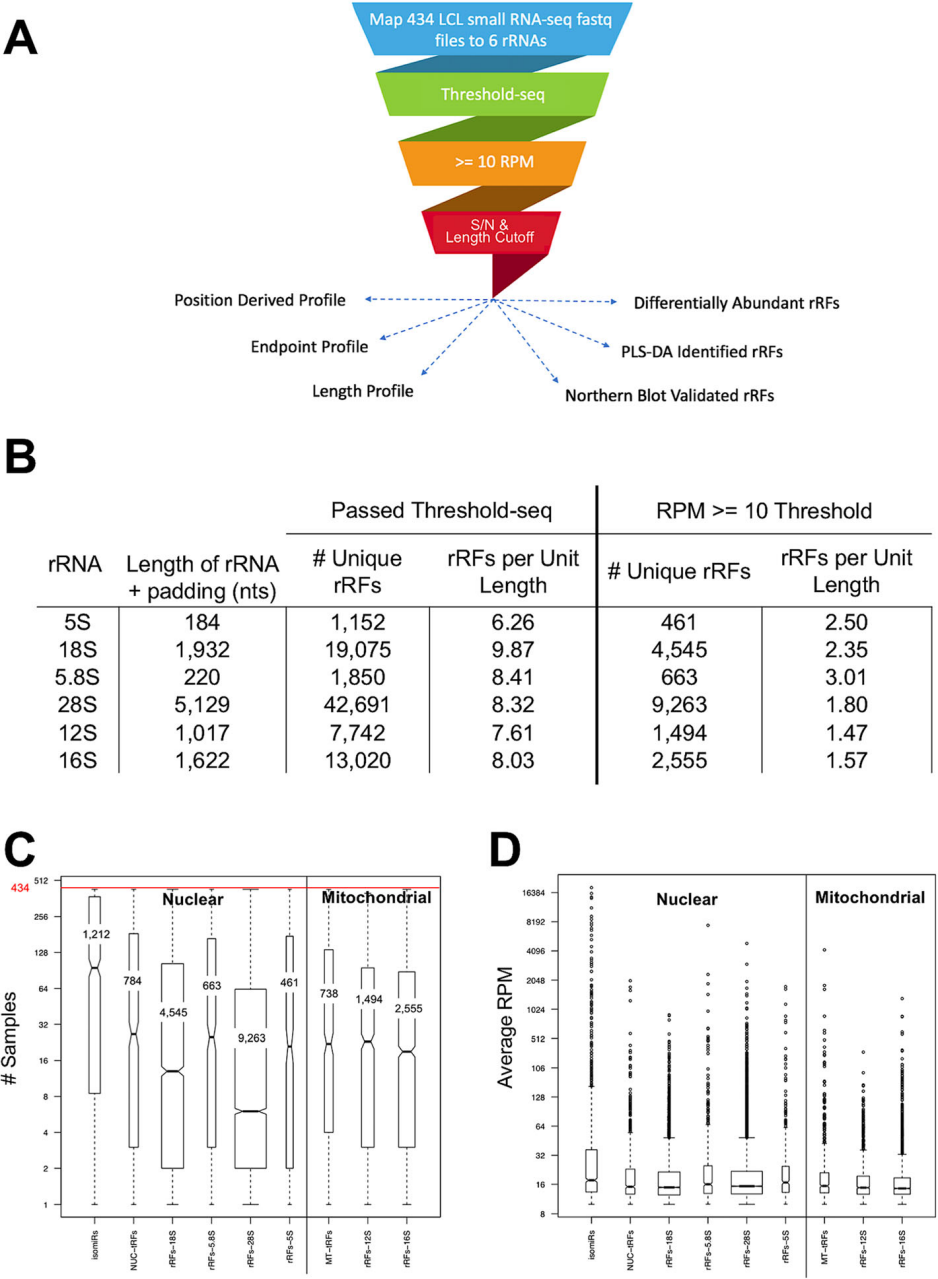
The rRF analysis pipeline reveals many unique fragments. **a** Workflow of the pipeline. **b** Table showing the number of unique rRFs that map to each rRNA and the number of rRFs per unit length, for the 434 LCL samples. The rRNAs were padded with 50 nts on each side. Note that many rRFs have abundance ≥ 10 RPM. **c** Distribution of the number of LCL samples (out of 434) in which a given isomiR, tRF, or rRF could be found at an abundance ≥ 10 RPM. The boxplots are grouped by genome of origin, nuclear or mitochondrial. **d** Boxplots show the distribution of the average abundance (in RPM) for molecules belonging to each category. Only molecules with abundance ≥ 10 RPM were considered in our subsequent analysis. Boxplots are grouped by genome of origin. **c**, **d** The width of the boxes is proportional to the number of unique molecules in each category, and the horizontal bars in each box represent the median RPM

After removing all samples that came from facility “six” (see the “Methods” section), there remained 434 RNA-seq datasets from the 1000 Genomes (1KG) Project [32] for our downstream analysis. The datasets represented individuals belonging to five population groups: Utah Residents with Northern and Western European Ancestry (CEU), Finnish in Finland (FIN), British in England and Scotland (GBR), Toscani in Italia (TSI), and Yoruba in Ibadan, Nigeria (YRI). In Fig. 1a, we present a pictorial summary of the pipeline that we used to analyze the 434 LCL datasets. The pipeline begins with a brute-force, deterministic, and exhaustive mapping of all sequenced reads on the 6 rRNAs. Only reads that matched the rRNAs exactly are kept. The pipeline also keeps track of rRFs that straddle either the 5′- or the 3′-end of the six reference rRNAs (see the “Methods” section). Any rRFs whose abundance does not satisfy a sample-specific threshold determined by Threshold-seq [35] (see the “Methods” section) are discarded. Figure 1b column 3 shows the number of unique rRFs produced from each rRNA that passed the Threshold-seq cutoff. In addition to Threshold-seq, we normalize each rRF’s abundance to reads-per-million (RPM), and for added stringency, we enforce a stricter minimum threshold of ≥ 10 RPM. Figure 1b column 5 shows the number of unique rRFs produced from each rRNA that pass the 10 RPM cutoff. During a final filtering stage, we discarded those rRFs whose instances outside of rRNA space exceeded 2% of all their genomic instances and a signal-to-noise ratio (S/N) ≥ 50 (see the “Methods” section).

### All six rRNAs produce many abundant short fragments

We find that all six rRNAs produce many abundant rRFs. Column 3 in Fig. 1b shows the numbers of unique rRFs from each rRNA that exceed the sample-specific adaptive threshold determined by the Threshold-seq algorithm [35] (see the “Methods” section) across the 434 analyzed datasets. Column 4 shows the same number normalized by the length of the parental rRNA. Also listed in the same panel are the numbers of unique rRFs (column 5) that map to each rRNA and exceed the threshold of 10 RPM. Column 6 shows the same numbers after they have been normalized by the different rRNA lengths. Only 18,981 rRFs survive this stringent threshold across all rRNAs. As can also be seen in Fig. 1b, across all six rRNAs, the number of rRFs per unit length range from 6.26 to 9.87, i.e., falls within a narrow window of values, which suggest that rRFs are processed at a consistent rate. For comparison purposes, we also examined isomiRs (using the same brute-force mapping as with the rRFs) and tRFs (using the MINTmap algorithm [36]) and computed their normalized RPM abundance. For the isomiRs, tRFs, and rRFs, we used as denominator the total number of sequenced reads of each sample. There were 1522 tRFs and 1212 isomiRs whose abundance was at least 10 RPM.

### Specific rRFs are present in all samples while others are group-specific

Considering that all 434 samples belong to the same cell type (immortalized B cells), we wanted to know if the rRFs that map to each of the six rRNAs recur across these samples. To this end, and separately for each rRF, we counted the number of samples (among the 434) in which each rRF exceeded the stringent threshold of 10 RPM. We combined the results for rRFs from the same rRNA into a single boxplot (Fig. 1c). For comparison purposes, we also generated the respective boxplots for the 1522 tRFs and 1212 isomiRs that also satisfy the threshold of 10 RPM. The resulting distributions are shown side by side in Fig. 1c. The width of each notched boxplot is proportional to the number of unique fragments within the respective type of short ncRNA; this number is also indicated in each of the boxplots.

Despite the fact that the samples represent a single cell type, the boxplots in Fig. 1c indicate that the bulk of the rRFs associated with each of the six rRNAs appear in only a fraction of the samples. The rRF distributions for 18S and 28S are particularly notable: while these two rRNAs produce many rRFs (4545 and 9263 respectively), most of them are present in only a small fraction of the 434 samples (Fig. 1c). This suggests a dependence on other variables, a point that we will be addressing below.

It is important to stress that these observations regarding rRF abundance are analogous to our previous findings for isomiRs and tRFs [23, 25, 26, 29], which are recapitulated by the respective distributions of Fig. 1c. For example, note how the typical isomiR exists in only 21.9% of the 434 samples (median = 95 samples). Of all present isomiRs, only a small number are present in most of the 434 samples. A similar observation can be made for the tRFs: here, again, only a very small number of tRFs appear in most of the 434 samples.

### rRFs are just as abundant as previously established types of ncRNAs

Next, we examined the abundance of rRFs across all 434 LCL datasets by juxtaposing their normalized abundances to those of isomiRs and tRFs (Fig. 1d). While the values of the median abundance across all three types of RNAs are essentially the same, it is worth noting that the abundances of the individual RNAs span a very wide range, from 10 to over 16,000 RPM with the rRFs spanning 10 to ~ 8000 RPM. Above, we mentioned that the number of unique rRFs depends on the parental rRNA (Fig. 1b). However, it is clear that the number of unique rRFs does not correlate with rRF abundance. Indeed, as can be seen from Fig. 1d, the rRFs and tRFs have similar abundance distributions even though their respective molecular categories comprise distinctly different numbers of fragments. Note also how all depicted categories have outliers with extremely high RPM values.

### Both the length and sequence composition of rRFs are relevant

In all of our analyses, we have been excluding sequenced reads shorter than 16 nucleotides (nts). This is because such short sequences that can map to, for example, a tRNA or an mRNA are also more likely to map elsewhere on the genome [37, 38]. This ambiguity makes it difficult to pinpoint the true genomic origin of the respective RNAs.

The 434 LCL samples contain many abundant fragments with lengths between 16 and 33 nts that map to the six reference rRNAs. To investigate which of these putative rRFs can also be found outside of the “rRNA space” (see the “Methods” section), we searched for each such sequence across the entire genome using a brute-force, deterministic approach. This allowed us to calculate for each *k*-mer the following “signal-to-noise” ratio (S/N) (see the “Methods” section): *number of instances the k-mer has inside rRNA space* over *number of instances the k-mer has outside of rRNA space*. Only *k*-mers that had an S/N ≥ 50 were considered further.

We found that the sequence composition of 5S rRFs is rather unique among the six rRNAs. Even 5S rRFs with only 16 nts have an S/N ≥ 63. The S/N for all combinations of length and parental rRNA source are listed in Table 1. As the table shows, the minimum length of the rRFs that satisfy this cutoff differs for each of the six rRNAs. The minimum lengths are as follows: 16 nts for 5S; 18 nts for 12S, 18S, and 5.8S; and 19 nts for 16S and 28S. Only rRFs that satisfied these minimum length cutoffs and whose corresponding S/N ≥ 50 were used in the subsequent analyses.

**Table 1:**
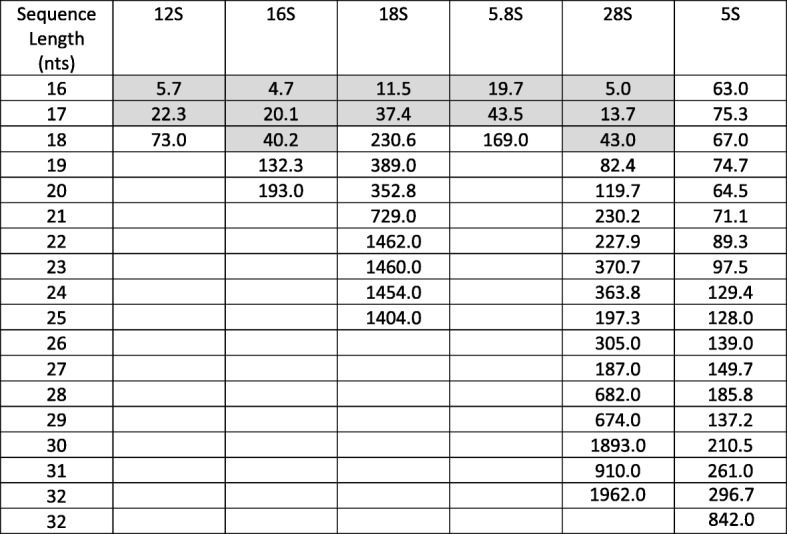
Signal-to-noise ratio. We define the ratio as “rRF instances inside rRNA space” over “rRF instances outside of rRNA space.” The value of S/N for each rRNA and rRF-length combination is shown

The shaded cells indicate length/rRNA combinations that do not pass our S/N ≥ 50 cutoff—the respective rRFs do not enter our analysis. The empty cells indicate that there are *no* fragments with these lengths outside of the rRNA space

### rRFs arise from “hotspots” within each rRNA’s span

Next, we sought to determine where the various rRFs map along each rRNA. We find that the rRFs can arise from any portion of the parental rRNA’s span. To be consistent with the notation that is used for tRFs [26–31, 36], we refer to those rRFs that arise from the 5′-end of an rRNA as “5′-rRFs,” those that arise from the interior of an rRNA as “i-rRFs,” those that arise from the 3′-end of an rRNA as “3′-rRFs,” and those that straddle the 5′- or 3′-ends of an rRNA as “x-rRFs.” The sequences of the 16,279 rRFs that survive the stringent thresholds (Threshold-seq, ≥ 10 RPM, length cutoffs) are listed in Additional file 4. For each rRF, we indicate its type (5′-rRF, i-rRF, 3′-rRF, or the terminus crossing x-rRF). We also list each rRF’s “license plate” extending to the rRFs the labeling scheme we introduced for the tRFs in 2016 [27] and have been using to label the tRFs that are currently in MINTbase [28]. The license plate labeling scheme guarantees a unique label for each rRF and *vice versa*, and is particularly suitable for labeling rRFs given the numerous copies that they have on the genome.

**Fig. 2:**
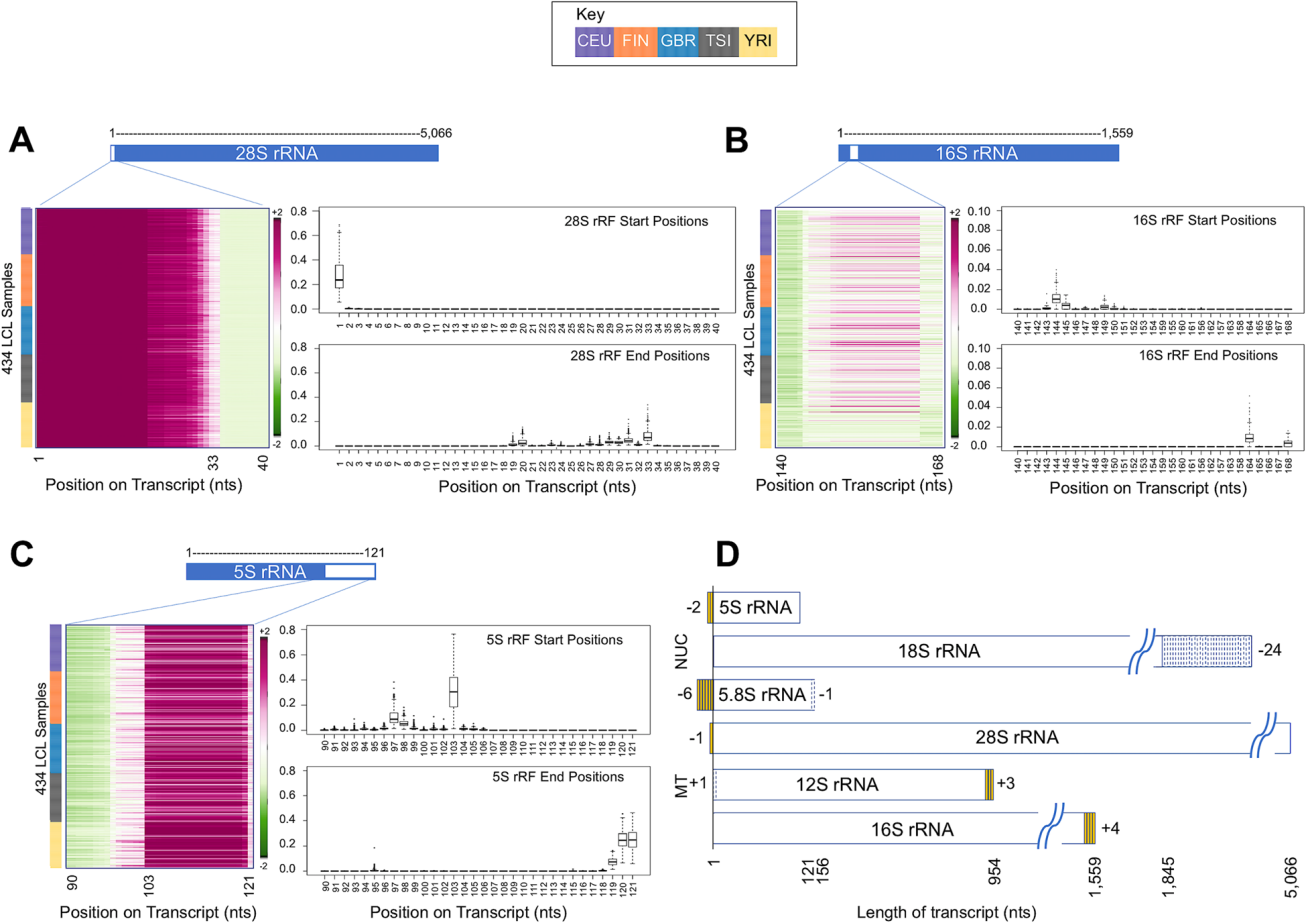
Examples of rRF-producing hotspots. **a**–**c** Shown are regions from 28S rRNA (**a**), 16S rRNA (**b**), and 5S rRNA (**c**). Each heatmap depicts the relative abundance of rRFs across all of the 434 LCL samples that map to the region of interest. Each adjacent boxplot displays the distribution of start and end positions of rRFs for the region shown in the heatmap. The heatmaps are scaled by row (sample). **d** Each reference rRNA (GenBank) is shown as a rectangle with solid blue contour. The solid black vertical line denotes position 1 of the reference rRNAs. Observed endpoints of x-rRFs that map immediately upstream or downstream of the reference rRNA sequences are represented by yellow boxes. Reference rRNA transcript locations that are adjacent to the nominal endpoints and did not have any rRFs mapping to them are shown with empty boxes with dashed lines. Not drawn to scale

The heatmaps in Fig. 2a–c show a few examples of the relative abundance of rRFs that map to highlighted regions of the 28S, 16S, and 5S rRNAs. In each case, we grouped samples from the same population into consecutive rows that we colored differently for each population: CEU—purple; FIN—orange; GBR—cyan; TSI—gray; and YRI—yellow. Adjacent to each heatmap are boxplots indicating the distributions of the starting and ending locations for the shown rRFs.

Several observations can be made readily. For instance, in Fig. 2a shows that the 28S produces 5′-rRFs that begin at position +1 in all 434 samples (all five populations and both sexes). Notably, in many of the 434 samples, the 28S rRNA also produces x-rRFs that begin at position -1 (data not shown). Another observation is that the 16S region shown in Fig. 2b produces i-rRFs preferentially in the four European populations (CEU, FIN, GBR, and TSI) but not in the African population (YRI).

The 3′-region of the 5S rRNA is shown in Fig. 2c. Many of the rRFs that map to this portion of the rRNA are 18 nts or 19 nts long. Of all the rRFs that map to the 5S rRNA, ~ 30% begin at position 103 and end primarily at either position 120 or position 121 (i-rRFs and 3′-rRFs respectively).

### The endpoints of rRFs do not always align with the endpoints of the parental rRNAs

Our analyses of the rRFs’ endpoints led to an intriguing observation. We found that a number of observed rRFs with abundance ≥ 10 RPM can either straddle the boundaries of several reference rRNAs or avoid them altogether (see the “Methods” section for reference identifiers). Figure 2d summarizes this observation. The solid-line rectangles denote the six rRNA transcripts. Positions that are present in the analyzed rRFs and correspond to positions beyond the rRNAs’ reference boundaries (whether upstream or downstream) are indicated in yellow, where the yellow boxes represent additional nts. Positions that are proximal to either the 5′ or 3′ termini of an rRNA and are not present in any of the analyzed rRFs are indicated with dashed lines, whereas the empty boxes represent the omission of nucleotides.

Several observations are worth making here. First, the rRFs produced from the 5.8S rRNA in the LCL begin 6 nts upstream from the nominal 5′-end listed in GenBank. Interestingly, it is known that there are two 5.8S isoforms: the shorter of the two is the one listed in the GenBank entry NR_145819.1 (see the “Methods” section) whereas the longer one extends a few nucleotides upstream (Fig. 2d). It was recently reported [19] that in previous studies, the shorter of the two isoforms was most abundant. While this may well be the case in LCL too—our analysis examined short and not long RNA-seq datasets—our data shows that the longer isoform produces the most 5′-rRFs. In addition to 5.8S transcript variants, there are rRFs produced from the 28S and 5S rRNAs that begin one and two nucleotides upstream of the rRNA’s nominal 5′-ends, respectively. The rRFs from 12S appear to *avoid* the first position of this rRNA: instead, all of them start at the second position. For 18S, none of the analyzed rRFs include any of the last 24 positions of the rRNA: indeed, the rightmost rRF terminates at position 1845 whereas the length of this rRNA is 1869 nts. Finally, we note that both 12S and 16S produce x-rRFs that straddle the respective annotated 3′ termini and borrow nucleotides from the downstream tRNA^ValTAC^ and tRNA^LeuTAA^, respectively.

### The lengths and abundances of the prevailing rRFs differ for each rRNA

**Fig. 3:**
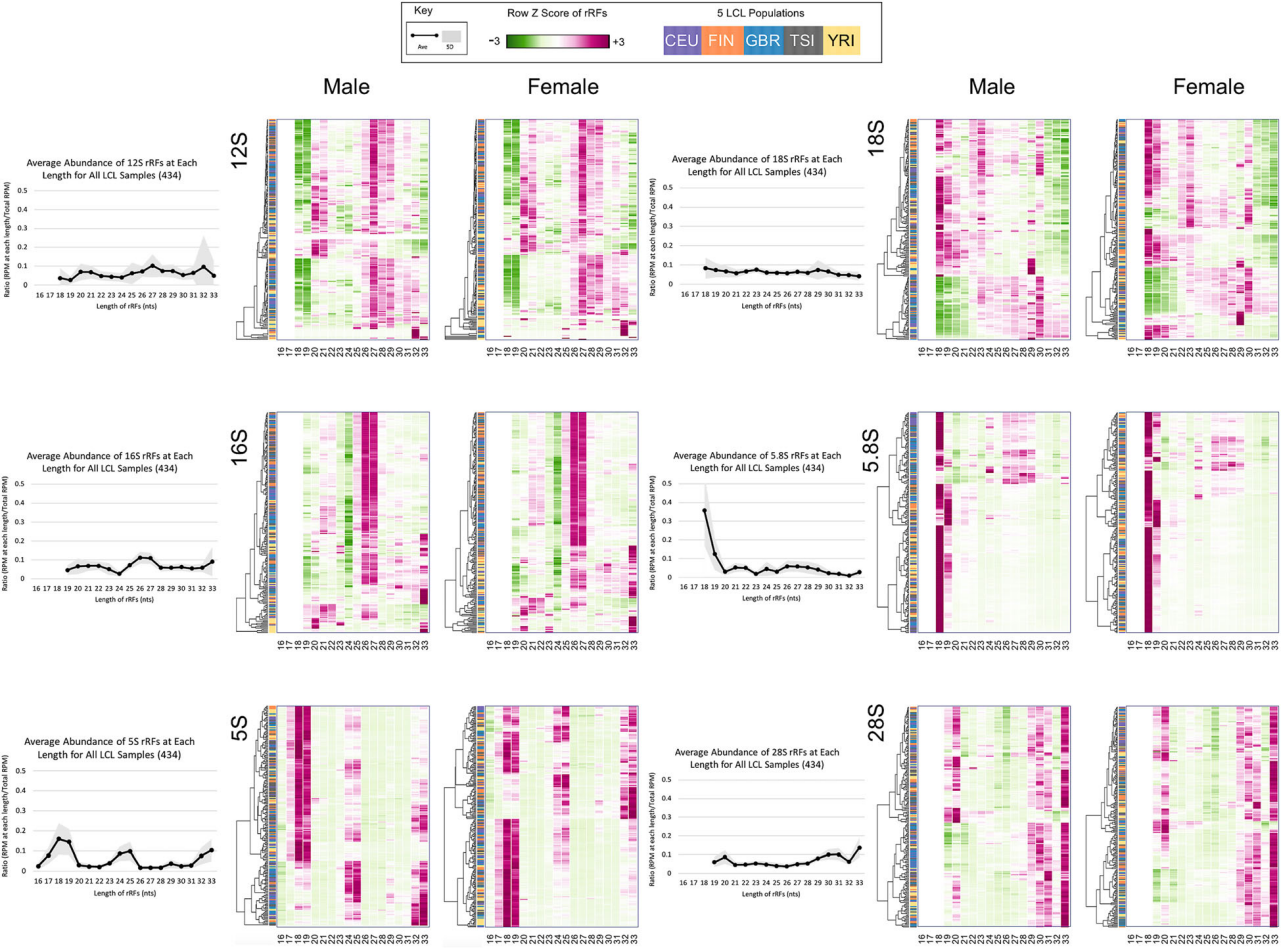
Length and abundance profiles of rRFs are globally recurring and population-specific. The line graphs show the ratio (RPM of each length/total RPM) for each length rRF and each of the 6 rRNAs where the black curve represents the average length ratio across all 434 LCL samples and the gray area represents the standard deviation of the ratio. Each heatmap shows the rRF length ratios at each sequence length, separately for the male (207) and female (227) samples. The heatmaps are hierarchically clustered by row. Dark green corresponds to the least abundance (*z-*score of − 3), and dark magenta to the most abundance (*z-* score of + 3)

We next sought to determine whether the lengths of rRFs are quantized. The line graphs in Fig. 3 show, separately for each of the six rRNAs, the length distributions of rRFs that survive the abundance, S/N, and length filters. As can be seen, the mapped rRFs have length profiles that are specific to each of the six rRNAs. The mitochondrial 12S and 16S rRNAs generate primarily longer rRFs (26–29 nts). In one third of the samples, 12S produces shorter (20–21 nt) rRFs too. The 5S rRNA produces short (18–19 nts) as well as intermediate (24–25 nts) and long rRFs (32–33 nts). However, note that in those samples where the short rRFs are prevalent, the intermediate and longer rRFs are generally absent, and *vice versa*.

The rRFs that are produced from the three 45S-derived rRNA (18S, 5.8S, and 28S) are rather intriguing. In terms of length, they span a wide range. 5.8S rRNA produces primarily rRFs with length 18 nts (35.7% of all rRFs map to this rRNA). 18S rRNA produces rRFs of all lengths. However, there is an evident bimodal behavior: in those samples where 18S rRFs with lengths between 18 and 23 nts inclusive are prevalent, longer rRFs with lengths 29 and 30 nt are absent, and *vice versa*. Lastly, very few rRFs with intermediate lengths (21–26 nts) map to 28S. Interestingly, in approximately one third of the samples, there are virtually no rRFs shorter than 28 nts that map to 28S. Below, we revisit these distributions by examining them separately for various subsets of the 434 samples.

### The length profiles of rRFs show similarities and differences across sexes and populations

By design, the 434 samples were selected to span two dimensions: sex and population origin. Specifically, the samples represent five population groups: of these, four groups are European populations (CEU, FIN, GBR, TSI) whereas the fifth group is an African population (YRI). Within a population, men and women are represented evenly. Figure 3 shows heatmaps of the distributions for the length of the various rRFs, separately for each sex and labeled by population. The color-coding scheme for the five populations is the same as in Fig. 2. The samples are hierarchically clustered using each sample’s length profiles. Overall, these heatmaps do not make apparent any strong dependence on sex or population origin. Nonetheless, careful inspection shows that such differences are indeed present.

As a matter of fact, YRI females produce consistently more 33-mers and fewer 26-/27-mers from 16S rRNA, compared to the other four populations, suggesting a population-specific signal that distinguishes between the European (CEU, FIN, GBR, TSI) populations and the African (YRI) population. On the other hand, European males produce consistently more 26-/27-mers than they do 33-mers.

### Numerous rRFs are differentially abundant by sex and population origin

**Fig. 4:**
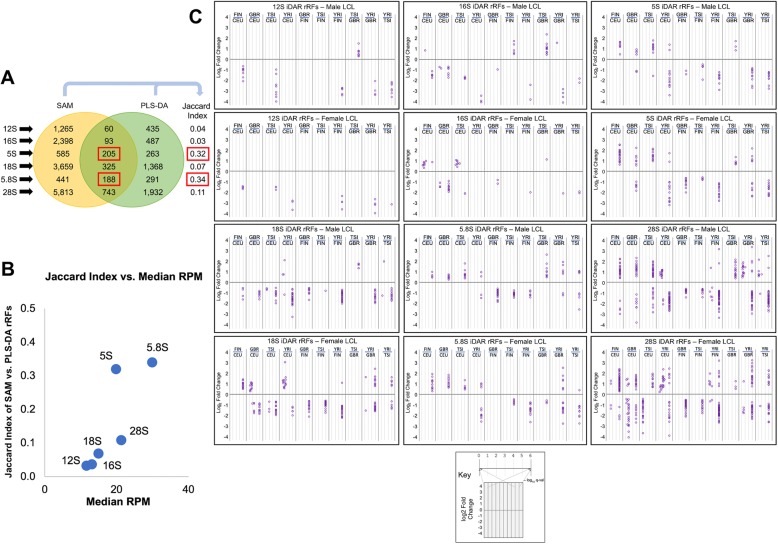
Numerous rRFs are differentially abundant by sex and population origin. **a** The number of instances where SAM identified an rRF as differentially abundant is shown in yellow (FDR ≤ 0.01). The number of instances where PLS-DA identified an rRF as differentially abundant is shown in green (VIP ≥ 1.5). The intersection of the two circles shows the number of comparisons where both SAM and PLS-DA found the same rRF to be differentially abundant. Also shown is the Jaccard index of the comparisons that are found to be differentially abundant by the two methods. **b** The Jaccard index for each rRNA is plotted against the median RPM of the iDARs. **c** For the iDARs, log_2_ fold change was plotted as a function of significance (*q*-value) for each of the 10 population combinations, and separately for males and females. The key at the bottom shows the *q*-value distribution in each of the 10 intervals that represent a population vs. population comparison

Even though the *lengths* of the rRFs mapping to the various rRNAs are largely consistent, we wanted to know if the *abundances* of these rRFs exhibit differences that are sex- or population-specific. To this end, we used two different approaches: SAM and PLS-DA (see the “Methods” section). At an FDR threshold of 0.01, SAM identified 3103 unique rRFs that are differentially abundant in at least one pairwise population comparison for samples belonging to the same sex. SAM also identified 88 unique rRFs that are differentially abundant by sex for samples belonging to the same population. The second method, PLS-DA, identified 1697 unique rRFs that are population-specific (same-sex samples compared) and 2264 unique rRFs that are sex-specific (same population samples compared). Figure 4c and Additional file 1: Figure S1A show the pairwise rRFs that are found by SAM and PLS-DA for each rRNA between all populations and the two sexes.

For stringency, we intersected the two collections of differentially abundant rRFs, which left us with 549 unique population-specific rRFs and 39 unique sex-specific rRFs (data not shown). We refer to the rRFs that both SAM and PLS-DA identify as differentially abundant in a given comparison as “the intersection of differentially abundant rRFs” or “iDARs.” By calculating the Jaccard index of the SAM-derived and PLS-DA-derived comparisons separately for each rRNA, we found that the 5S and 5.8S rRFs have the highest indices (0.32 and 0.34, respectively) for the *population-specific* pairwise comparisons (Fig. 4a). 5.8S also has the highest Jaccard index (0.17) for the *sex-specific* pairwise comparisons (Additional file 1: Figure S1A). Figure 4b shows that 5.8S produces iDARs with high median RPM (31.4) that also participate in comparisons with the highest Jaccard index (0.34) - shown with red box in Figure 4a. Meanwhile, 5S produces iDARs with the third highest median RPM (19.4) that also participate in comparisons with the second highest Jaccard index (0.32) - shown with red box in Figure 4a. Additionally, Figure S1B shows that the sex-specific iDARs from 5.8S have the second highest median RPM (48.4) and participate in comparisons with the highest Jaccard index (0.17). Together, this suggests that rRFs from the 5.8S rRNA can be population-specific and sex-specific.

When we look at the differential abundance of the iDARs, the acute differences by sex and population origin become readily apparent (Fig. 4c, Additional file 1: Figure S1C). To visualize the fold differences of the iDARs, we plotted the log_2_ fold change for all pairwise comparisons and separately for each sex (Fig. 4c). A quick glance at this panel reveals a striking commonality: regardless of how many rRFs are iDARs in each case, all six rRNAs produce many rRFs with consistently higher abundance in the four European populations (CEU, FIN, GBR, and TSI) than the African population (YRI). This holds true for both males and females.

Moreover, within the four European populations, there are population-specific differences. For example, the CEU population produces rRFs from the 5S and 5.8S rRNA in lower abundance than the FIN, GBR, and TSI populations, in both males and females. CEU males produce more 18S rRFs than their FIN, GBR, and TSI counterparts whereas CEU females produce variable 18S rRFs as compared to the other European females (FIN, GBR, and TSI). Interestingly, both males and females from the CEU population produce multiple “upregulated” and “downregulated” 28S rRFs as compared to the other four populations (FIN, GBR, TSI, and YRI).

We also looked in more depth at the differential abundance of the 88 unique, sex-specific iDARs (Additional file 1: Figure S1). Notably, almost all of these rRFs exhibit higher abundance in females than in males. The majority of these rRFs map to the 5.8S rRNA (16 unique iDARs). Surprisingly, the 5.8S rRFs exhibit sex-specific abundance differences only in three of the four European populations: CEU, GBR, and TSI. FIN and YRI females produce more iDARs from 16S, 18S, and 5S than males. And the one 28S iDAR that is sex-specific is present at a higher level in males than females in the GBR population.

### Sex- and population-specific differences in rRF abundance are group-defining

**Fig. 5:**
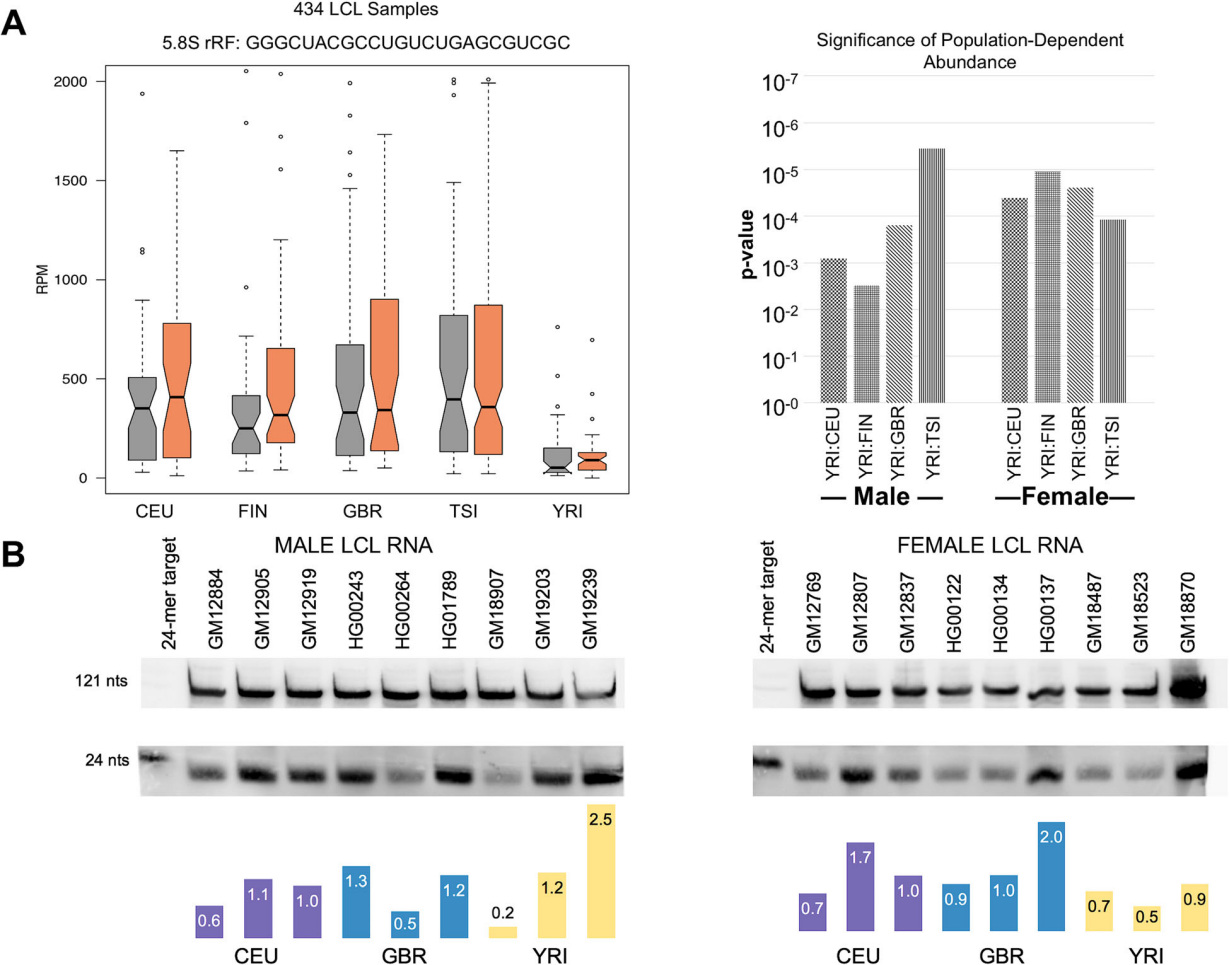
Independent experimentation validates the presence of rRFs in LCLs. **a** Boxplots show the abundance of the 5.8S 24-mer i-rRF (GGGCUACGCCUGUCUGAGCGUCGC) across the five LCL populations (434 total samples). Males are represented by gray boxes, and females are represented by orange boxes. The abundance is shown in the boxplots where there is a statistically significant difference between each population according to Welch’s *t*-test. **b** Two northern blots probing for the 5.8S 24-mer i-rRF in RNA from nine male and nine female LCLs. Three cell lines from each of CEU, GBR, and YRI were used for each sex. Each lane contains 5 μg of RNA from each sample, and the first lane in each blot has 5 pmol of 5.8S 24-mer target cDNA (lower band). Full-length 5S (ACGUCUGAUCUGAGGUCGCGU) is the top band and served as loading control. rRF bands were normalized to the 121-nt 5S loading control band. Below each blot is a quantification of the 5.8S rRF probe where CEU is represented by purple, GBR is represented by cyan, and YRI is represented by yellow

The rRF with the highest change in abundance is the 24-mer GGGCUACGCCUGUCUGAGCGUCGC from 5.8S. This i-rRF maps to the 3′-prime end of the 5.8S rRNA (Additional file 2: Figure S2) and ends two nucleotides shy of the nominal 5.8S transcript. This i-rRF is differentially abundant in the following comparisons: YRI/CEU (− 2.31 log_2_ fold change), YRI/FIN (− 2.37 log_2_ fold change), YRI/GBR (− 2.49 log_2_ fold change), and YRI/TSI (− 2.47 log_2_ fold change)*.* In Fig. 5a, the abundance of this 24-mer i-rRF is stratified by the boxplots. We observe the statistically significant trend which shows that all 4 European (CEU, FIN, GBR, and TSI) populations, independent of sex, produce more of this fragment than males and females from the YRI population. Because this i-rRF is differentially abundant between the YRI and all four European populations, the rRF may reflect geography-based differences at the level of a continent (i.e., European vs. African rRFs).

We also observe that the 5.8S i-rRF 19-mer UAAUGUGAAUUGCAGGACA, which is produced from the center of the 5.8S rRNA transcript, is differentially abundant (*p-*value = 0.03) by sex (Additional file 3: Figure S3A top), suggesting that it is potentially a sex-specific rRF. In addition to the significant sex-specific difference, we unpacked the sexes by population and observed that while there is an overall sex-specific trend, there is a strong population-specific dependence as well (Additional file 3: Figure S3A bottom) with CEU and GBR having statistically significant differences between the sexes with *p*-values of 0.014 and 0.010, respectively. Interestingly, while the CEU, GBR, and TSI females produce *more* of this 19-mer i-rRF than their respective males, FIN females actually produce *less* of this fragment than FIN males. YRI males and females produce the fragment at approximately the same abundance. We also investigated the abundance of a 5.8S 21-mer i-rRF UAAUGAGAAUUGCAGGACACA, which has the same 5′-end start position as the 19-mer i-rRF but contains two additional nucleotides on the 3′-end of UAAUGUGAAUUGCAGGACA (Additional file 3: Figure S3). Interestingly, we observed that just like the 19-mer, the 21-mer i-rRF is differentially abundant (*p*-value = 0.007) and maintains similar population- and sex-specific differences. However, this i-rRF is present at a lower abundance than the 19-mer i-rRF suggesting selective sequence production.

### Analyses of independently obtained samples corroborate the presence of rRFs in LCLs

In Fig. 1c, we saw that only a small number of rRFs, isomiRs, and tRFs are present in all LCL samples, suggesting fragment specificity based on subgroups. In addition, Fig. 5a shows that even while the differential abundance of individual rRFs is significant between populations, they still exhibit large “in group” abundance variations. With this in mind, we pursued experimentally the sex- and population-specific findings with the help of LCLs obtained from 18 healthy people from the 1KG Project which accounted for three populations (CEU, GBR, and YRI) (see the “Methods” section for the cell line identifiers). These 18 samples are *not* among the samples that were sequenced by the 1KG Project.

We ran northern blots with RNA from these cell lines using the reverse complement of the 5.8S 24-mer i-rRF GGGCUACGCCUGUCUGAGCGUCGC as a probe. The northern blots of Fig. 5b provide independent experimental validation of the presence of this i-rRF. There is a wide distribution of abundances across the 18 samples, which include samples from males and females (Fig. 5b). Note how this distribution is concordant with the findings of our computational analyses (Fig. 5a): indeed, while the median abundance of this i-rRF is characteristically and significantly higher for the four European populations compared to the African population, the actual abundance values span a very wide range within each population. Although we do not expect the difference of the median values to be captured by the handful of samples that we analyzed, we are able to see sex- and population-specific trends. The quantification of the northern blots in Fig. 5b shows that overall CEU and GBR females produce more of the fragment than their male counterparts. YRI females produce a similar amount of the i-rRF at a low abundance. Additionally, a population-specific difference trend between the two female European populations (CEU and GBR) and the one female African population (YRI) is discernible (Fig. 5b bottom). This trend cannot be observed among the few male samples we assessed.

### The identity and abundance profiles of rRFs can also differ across tissues

**Fig. 6:**
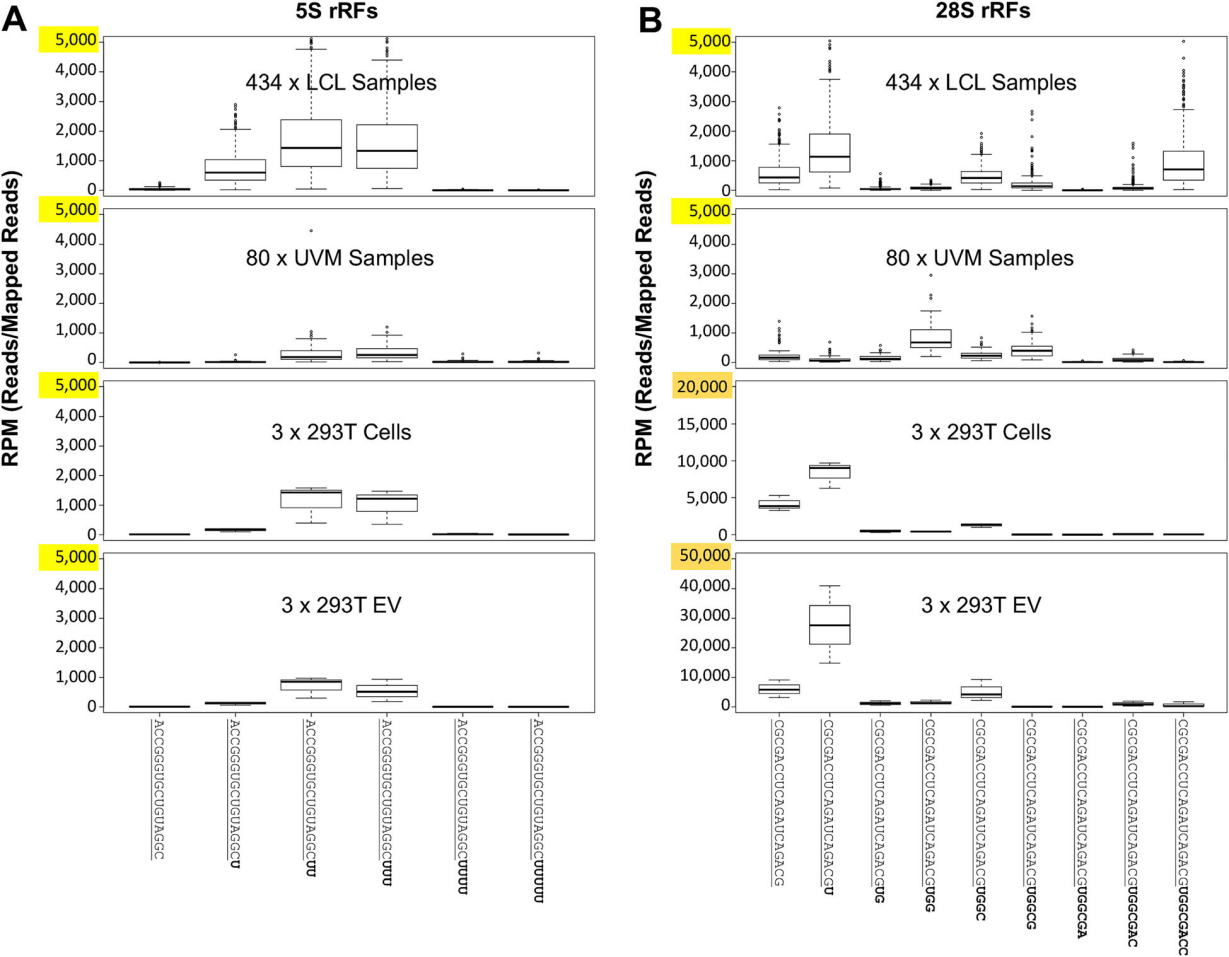
Preliminary evidence that the rRF profiles are tissue-dependent. Boxplots show the abundance of rRFs at each length for the 434 LCL samples, all 80 TCGA UVM samples, three replicates of 293T cells, and three replicates of 293T EV. Yellow boxes indicate the max *y*-axis values represented on each boxplot. The sequence that is common to all rRFs is shown underlined. Each rRF differs from its adjacent rRF by one nucleotide. **a** i-, 3′-, and x-rRFs produced from the 3′-end of the 5S rRNA transcript. **b** 5′-rRFs produced from the 28S rRNA transcript. The data values for the 5S and 28S rRFs for the three 293T cells and three 293T EV are in Additional file 4

We next wanted to know if the most ubiquitously and highly abundant rRFs we observe in the 434 LCL datasets also exist in other human tissues. For this purpose, we chose 80 RNA-seq datasets from primary uveal melanoma (UVM) samples from The Cancer Genome Atlas (TCGA) [33], as well as three 293T cell RNA-seq datasets and their three corresponding 293T EV RNA-seq datatasets from publicly available data from the Gene Expression Omnibus (GEO) [34] (see the “Methods” section and Additional file 4). Figure 6 tracks the most abundant rRFs from the LCL datasets in the other three collections. The most abundant rRFs, ACCGGGUGCUGUAGGCUU an i-rRF and ACCGGGUGCUGUAGGCUUU a 3′-rRF, come from the 3′-end of the 5S rRNA and differ by a single nucleotide. These i- and 3′-rRFs have lengths of 18 nts and 19 nts, respectively, and a median abundance of 1434 and 1334, RPM respectively. As can be seen from Fig. 6a, rRFs that correspond to shorter or longer instances of these two abundant rRFs are produced either at a lower abundance or not at all in the LCL collection. The UVM and 293T cell and 293T EV datasets show a similar distribution pattern to the LCL datasets. However, the abundance of these two rRFs is considerably lower in the 293T EV and UVM datasets. Moreover, neither the UVM nor the 293T cell or 293T EV datasets contain the 17-mer i-rRF ACCGGGUGCUGUAGGCU in any notable abundance.

Figure 6b tracks CGCGACCUCAGAUCAGACGU and CGCGACCUCAGAUCAGACGUGGCGACC, two 5'-rRFs from the 28S rRNA—their common sequence segment is shown underlined. The 5'-rRFs have lengths 20 and 27 nts, respectively, and are highly abundant in the LCL datasets (median abundances are 1136 and 707 RPM, respectively). In the UVM datasets, these 5'-rRFs are not very abundant. However, the abundance of the 22-mer variant CGCGACCUCAGAUCAGACGUGG is 676 RPM. Interestingly, not only do the 293T cells and 293T EV produce the 20-mer CGCGACCUCAGAUCAGACGU at an extremely high abundance (median abundances are 9030 and 27,593 RPM, respectively) but the 19-mer CGCGACCUCAGAUCAGACG is also highly abundant (median abundances are 3870 and 5807 RPM, respectively—note the different range of the *y*-axis in these two plots). Additionally, the 293T cells produce the 23-mer CGCGACCUCAGAUCAGACGUGGC at a median abundance of 1376 RPM whereas in the 293T EV the same 23-mer is present at an even higher median abundance (4192 RPM), which suggests that it is preferentially secreted. This 23-mer 5'-rRF is less abundant in the UVM and LCL collections.

### Persistence of rRF profiles across laboratories and different library preparation methods

It is conceivable that the differences in rRFs that we showed exist among the LCL datasets result from differences in the collection or growth protocols, or interlaboratory differences. The Geuvadis Consortium, which generated the datasets that we analyzed, selected five samples (one from each of the five populations) that they sequenced independently at seven different European sequencing centers [39], generating a total of 35 datasets. When we compare the 35 datasets using the top 1000 rRFs from each rRNA, we find high correlations across the sequencing centers (Additional file 5: Figure S4A).

It is also possible that the rRF differences we see are artifacts of cDNA library preparation. To address these possibilities, we deep-sequenced two age-matched and sex-matched, commercially available LCLs (that were not a part of the 1KG Project samples whose RNA-seq profiles were reported in the literature): ND02672, which is derived from a 63-year-old male African American, and ND07114, which is derived from a 66-year-old male Caucasian American. We deep-sequenced each cell line using two different library preparation kits: Illumina’s TruSeq and NEB’s NEBNext (see the “Methods” section). In each of the four datasets, we identified rRFs using the methodology described in our manuscript. For each of the six rRNAs, we identified the top 1000 most-abundant rRFs and used them to compute pairwise Pearson correlations (Additional file 5: Figure S4B). As can be observed in Additional file 5: Figure S4B, the inter-kit comparisons exhibit high correlations (Pearson correlation ≥ 0.76) indicating that the same rRFs were being identified by the two kits and with comparable abundances.

### Further evidence and presence of rRFs in the context of parental rRNA structures

In order to further validate our findings of persistent, highly abundant, and differentially abundant rRFs, we show northern blots for four rRFs that are abundant in the LCL datasets (Additional file 6: Figure S5): GGGCUACGCCUGUCUGAGCGUCGC (5.8S 24-mer i-rRF, average RPM = 489), UACGCCUGUCUGAGCGUCGCU (5.8S 21-mer i-rRF, average RPM = 642), ACCGGGUGCUGUAGGCUU (5S 18-mer i-rRF, average RPM = 1752), and CGCGACCUCAGAUCAGACGU (28S 20-mer 5′-rRF, average RPM = 1473) (see the “Methods” section). These include the 24-mer i-rRF that was differentially abundant (Fig. 5) as well as three new rRFs. Again, rRFs were compared to their respective synthetic DNA positive controls. We see in the northern blots in Additional file 6: Figure S5A-D that the rRFs for which we probed are part of a larger collection of distinct rRFs. Interestingly, the 5.8S 24-mer and 21-mer and the 5S 18-mer i-rRFs are strongly detected even in the context of the longer fragments whereas the 28S 20-mer is much less abundant when compared to the longer 28S fragments.

Next, we wanted to know whether these short rRNA fragments had their own secondary structure, which could suggest potential biogenesis mechanisms. In Additional file 6: Figure S5, we see that two of the three 3′-rRFs have consistent hairpin structures, while the remaining one is partially structured and the 28S 5′-rRF has no structure at all. We also examined the location of the four rRFs in the predicted secondary structure of the full-length rRNAs (Additional file 7: Figure S6). We observe that while the two 5.8S i-rRFs (Additional file 7: Figure S6A) map to the same overall location of the parental 5.8S rRNA, they are being processed from different structural locations. The 24-mer 5.8S i-rRF is cleaved at its 5′-end in the middle of a double-stranded hairpin structure of the 5.8S rRNA (between position 131-132) while its 3′-end is two nucleotides shy of the 3′-end of the full-length rRNA (position 155). The 5′-end of the 21-mer 5.8S i-rRF is cleaved right at the intersection of two RNA hairpins (position 136) and extends to the second to last position of the 3′-end of the full-length 5.8S rRNA (position 156). In Additional file 7: Figure S6B, we can observe that the 5′-end of the 18-mer 5S i-rRF is cleaved at the base of a loop (between position 102-103) and extends to the second to last nucleotide in the full-length 5S rRNA (position 120). Finally, in Additional file 7: Figure S6C, the 20-mer 28S 5′-rRF has its 3′-end at the base of a loop (position 20).

Taken together, the findings and results shown in Figs. 2, 3, and 5 suggest that human rRFs are produced from specific hotspots that persist across cell types. However, these hotspots produce rRFs with endpoints, lengths, and abundances that suggest dependence on cell type and tissue type, in addition to the dependence on sex and population origin that we discussed above. Moreover, these RNAs appear to be packaged into extracellular vesicles selectively. These rRF properties mirror previously-reported findings on isomiRs [20, 23, 25, 29, 40–43] and tRFs [21, 22, 26–31, 36, 44–51].

## Discussion

The early discoveries of short ncRNAs were first based on forward genetics with a phenotype motivating the discovery of a genomic or transcriptomic cause. Following the surge of NGS, short and long ncRNAs are being continuously discovered via reverse genetics and in an unbiased manner. In fact, NGS has allowed the discovery of novel categories of ncRNAs and the enumeration of their members. While short ncRNAs such as miRNAs, isomiRs, tRFs, snoRNAs, and piRNAs have been studied extensively in health and disease for more than a decade already [52], short ncRNAs that derive from rRNAs have been largely overlooked. Arguably, this is because of their high abundance in cells where full-length rRNAs comprise about 80% of all RNA molecules present and are actively removed from long sequencing data using commercial methods like TruSeq Stranded Total RNA Gold (Illumina) and RiboMinus (ThermoFisher). While the removal of rRNAs is routine in long RNA-seq (where one wishes to quantify mRNAs and long ncRNAs), rRNA depletion is not part of the short RNA-sequencing protocols. Presumably, this is because rRFs are nowhere as abundant in the cell as the full-length rRNAs. This led to early reports of rRFs. The availability of many datasets from the same tissue or cell type has now made possible comparative studies such as the one we presented here, and the increasing accumulation of evidence that rRFs comprise a molecular category that warrants in-depth exploration [19].

In this study, we reported our findings on the rRFs, the emerging class of short fragments that derive from nuclear and mitochondrial rRNAs. The rRFs arise from all six reference rRNAs: the four nuclear rRNAs (18S, 5.8S, 28S, 5S) and the two MT rRNAs (12S, 16S). To characterize rRFs, we designed and used a pipeline that applied stringent filters aimed at discarding putative rRFs that are either not adequately abundant or unlikely to arise from the six rRNAs. Application of the pipeline to 434 short RNA-seq datasets from the 1KG Project identified numerous unique sequences that are derived from these six rRNAs. These sequences are present at abundance levels that parallel those of isomiRs and tRFs (Fig. 1), which is a first indication that rRFs could be important in analogy to isomiRs [20, 23, 25, 29, 40–43] and to tRFs [21, 22, 26–31, 36, 44–51].

A key consideration of our approach was to account for the unique genomic attributes of rRNAs. As mentioned in the Background, rRNA genes have many genomic copies each. At the same time, and in addition to the full-length rRNAs, the nuclear genome is riddled with numerous partial copies of rRNAs. This is in parallel to what we observed for tRFs [26, 30, 31, 48]. However, unlike tRFs, the partial copies of the longer rRNAs such as 12S, 16S, 18S, and 28S that can be found on the genome are themselves long. The copies also have extensive sequence similarities with the original rRNA templates. It is difficult to argue against the possibility that these retained partial copies have been co-opted into new roles in the cell. Consequently, and unlike what we did in the case of tRFs and tRNA space, we defined the “rRNA space” as the union of all full-length rRNAs and of all partial copies found in the nuclear genome that were at least 16 nts long.

We also found that within the rRNA space, sequence composition matters. For example, even short rRFs, e.g., 16-mers and 17-mers, that map to 5S are highly unlikely to be found elsewhere on the genome (high S/N). However, this is not the case for 16-mers and 17-mers that map to the remaining five rRNAs. We accounted for these differences by evaluating rRFs of all lengths and separately for each rRNA. This allowed us to establish for each rRNA a minimum length at which its rRFs have an S/N ≥ 50 (Table 1). By enforcing this minimum S/N cutoff, we discarded shorter rRFs that map outside of rRNA space with high frequency.

Our analyses revealed that highly abundant rRFs are produced from specific “hotspots” that are different for each of the six rRNAs (Fig. 2). Additionally, the rRFs have distinct starting and ending points, and favor specific lengths (Fig. 3), which are also specific to each rRNA. Despite these differences, the rRF profiles remain largely unchanged across like samples (Fig. 2 and 3). These observations argue in support of a regimented process that underlies the biogenesis of rRFs and differs for each rRNA.

During the mapping of sequenced reads to rRNAs, we also kept track of instances where the mapped reads might straddle the genomic boundaries of the reference rRNAs. This allowed us to discover multiple instances of rRFs that straddle the nominal endpoints of rRNAs (Fig. 2d). We believe that this is an important observation. For example, if we do not allow reads to straddle the boundaries of rRNAs, we find no reads mapping on the 5′-end of 5.8S rRNA. Similarly, no reads would have been mapped on the 3′-end of 16S rRNA. Given that these observations persist across so many biological samples, it is reasonable to assume that these rRFs play important roles in the cell. Consequently, it will be prudent for future studies to continue to consider rRFs that straddle the known boundaries of rRNAs.

We also examined the possible dependence of rRF profiles on sex and population origin. We found statistically significant differences in the rRF profiles of datasets that differed by sex, population origin, or geographical ancestry (Fig. 4). In our computational analysis, we had a lot of statistical power due to the 434 samples we were analyzing (~ 40 female and ~ 40 male samples for each population group) and the population-specific differences were clear despite the wide range of rRF abundances within a population group (Fig. 5a). Even though we were limited by six LCL samples from each population and three LCL samples for each sex, we were able to recapitulate the variability of the abundance of the 24-mer 5.8S i-rRF and observe trends supporting the i-rRF’s dependence on sex and population (Fig. 5b).

Our analyses also provide preliminary evidence suggesting that rRF fragmentation profiles change as a function of tissue type. We saw that for select rRFs, the relative abundance changed when we compared the LCL, UVM, 293T cell, and 293T EV datasets (Fig. 6). In addition, we observed the importance of rRF composition in tissue type. For example, while a 20-mer 5′-rRF is highly abundant in the LCLs, 293T cells, and 293T EV, it is absent in the UVM samples. Furthermore, a 5′-rRF with 2 additional nucleotides is absent in the very same LCL, 293T cell, and 293T EV samples and abundant in the UVM samples (Fig. 6). This tissue-dependence observation is something that we showed to be the case through two large-scale analyses of isomiRs [25] and tRFs [30]. And, a recent report in bioRχiv [53] showed evidence that the aggregate production of rRFs from the 5′-end of 28S differs across several tissues. We note, however, that this last study examined the wholesale rRF production from the 5′-end of this rRNA and did not show how specific rRFs changed in abundance across tissues.

Had rRFs been degradation products, one would have expected to see them scattered across the length of the various parental rRNAs. Moreover, their 5′ and 3′ endpoints would not be expected to show preferences for any particular position [54]. Perhaps more importantly, the stochastic nature of the process would mean that the relative abundance of any two rRFs from the same or different parental rRNAs would not be expected to remain constant across samples. However, what we observe is a combination of two things: a persistent preference for specific endpoints in like samples and abundance ratios that remain constant in like samples (Figs. 3 and 4).

In this study, we also provide evidence that rRFs are not the product of technical variability. By comparing, deep-sequencing data for the same five LCLs that were generated by seven different sequencing centers, we found high inter-center Pearson correlations when we analyzed the top 1000 rRFs of each replicate (Additional file 5: Figure S4A.) We also observed that independent of the cDNA library preparation kit (Illumina’s TruSeq or NEB’s NEBNext), the resulting deep sequencing generated very similar rRF profiles for the same cell line. This was indicated by the high value of the pairwise correlations that we computed by using the top 1000 rRFs (Additional file 5: Figure S4B).

We validated the presence of rRFs that our computations determined to be important. We ran northern blots and probed for 4 different rRFs in LCLs that were not part of the sequencing data analyzed for this study (Additional file 6: Figure S5). We were able to detect these fragments as well as additional longer fragments with distinct lengths. For example, as shown in Additional file 6: Figure S5D, while we are able to detect the smaller 20-mer 28S 5′-rRF, we see several mid-length rRFs. Because the LCL sequencing data we analyzed contained reads up to 33 nt, any fragments that were longer would not have been present in the sequencing data that we analyzed. Future rRF research should consider this when designing short RNA-seq experiments.

Furthermore, as Additional file 6: Figure S5A shows, there are several rRFs with similar core sequences that can be detected by a given northern probe (e.g., GGGCTACGCCTGTCTGAGCGTCGC and TACGCCTGTCTGAGCGTCGCT share a core sequence). While this confirms the presence of several more rRFs in addition to the rRF being sought, it does not necessarily help determine the identity of these other rRFs. Complicating matters is the fact that with the exception of deep sequencing, there are no other *commercially available* schemes that can measure the amount of a specific short RNA (e.g., isomiR, tRF, or rRF) while guaranteeing the identity of both of its endpoints [55]. The recently published dumbbell-PCR method [56] is a very effective and innovative solution to the quantification problem but is not scalable. It is worth mentioning here that while deep sequencing is effective in detecting and measuring rRFs, it also has a few limitations. For example, in its standard version, it will only detect and report RNAs that have 5′-P and 3′-OH endpoints, respectively. Consequently, abundant non-conforming RNAs will not be reported without additional considerations [57]. Additionally, just like their rRNA precursors, rRFs are expected to carry nucleoside modifications that can potentially interfere with the reverse transcription step of cDNA library preparation. Whenever this occurs, and since cDNA library preparation relies on the ligation of 5′ and 3′ adapters to the present RNAs prior to amplification, the corresponding rRFs will not get amplified and thus will not be among the sequenced reads [58]. In other words, it is likely that the true complement of rRFs that are present in a cell is a superset of what we can identify and report by analyzing collections such as the one we discuss in this presentation. We also note here a related analysis where we additionally examined whether the modifications whose locations within tRNAs are known could give rise to artificially produced tRFs: our analyses of tRFs found in more than 10,000 TCGA datasets representing 32 cancers and multiple tissues do not show any evidence that this is the case [30].

It is important to stress that these are but nascent studies of a new category of short ncRNAs whose biogenesis and functional roles elude us currently. The findings bear notable similarities to the dependencies we have been reporting for two other large categories of short ncRNAs, the isomiRs [20, 23–25, 29, 40–43] and the tRFs [21, 22, 26–31, 36, 44–51]. Additionally, there is a clear and recurrent consistency in both health and disease settings and a dependence on a person’s attributes [25, 30]. The work that we presented above is adding to emerging evidence in support of further studies aimed at uncovering the roles of these molecules in the cell. Knowing which rRFs are differentially abundant between which groups of samples (whether the groups are defined by sex, population of origin, or other variable) can help prioritize among these new molecules and focus subsequent work.

## Conclusion

These findings on rRFs add to the continuously growing and extremely important small ncRNA field. In conclusion, our analysis shows rRFs are uniquely produced, highly abundant, and context-specific, thus providing a comprehensive scaffold to build future work in areas of biogenesis, function, disease biomarkers, and other elements of RNA biology.

## Methods

### Dataset: 1000 Genomes Project

We used the short RNA-seq datasets that were released by the 1KG Project [32] and were derived from the lymphoblastoid cell lines (LCL) of individuals belonging to five population groups: CEU (Utah Residents with Northern and Western European Ancestry), FIN (Finnish in Finland), GBR (British from England and Scotland), TSI (Toscani in Italia), and YRI (Yoruba in Ibadan, Nigeria). The 1KG Project released 452 total short RNA-sequencing datasets (and 35 technical replicates). The samples were sequenced at seven facilities (Geuvadis Consortium). The 48 samples that were sent to one of the facilities (number “six”) were sequenced using 47 cycles of sequencing whereas all other samples were sequenced using 33 cycles. In order to be consistent, we removed all samples that came from facility “six”—this left us with 434 LCL datasets for our downstream analyses (83 CEU, 94 FIN, 88 GBR, 87 TSI, and 82 YRI). We used the 35 technical replicates (1 CEU, 1 FIN, 1 GBR, 1 TSI, and 1 YRI sequenced at seven facilities) for the rRF correlations.

### Other datasets: Gene Expression Omnibus and The Cancer Genome Atlas

We analyzed short RNA-seq for six samples from the Gene Expression Omnibus (GEO) data (GSE99430) [34] which looked at the 293T cells and their derived extracellular vesicles (EV). 293T cell samples are as follows: SRR5628228, SRR5628229, and SRR5628230. The EV samples are as follows: SRR5628231, SRR5628232, and SRR5628233. The abundances of the rRFs found in these datasets can be found in Additional file 4. We also analyzed short RNA-seq data for the 80 uveal melanoma (UVM) samples from The Cancer Genome Atlas (TCGA) [33].

### Reference rRNAs

We used the GenBank 45S (RNA45SN1), 5S (RNA5S12), 12S (MT-RNR1), and 16S (MT-RNR2) rRNAs as our reference rRNA sequences for this analysis. The GenBank accession numbers are as follows: NR_145819.1, NR_023374.1, NR_137294.1, and NR_137295.1, respectively. RNA45SN1 was chosen as a representative 45S and is 13,351 nucleotides (nts) long. RNA5S12 was chosen as a representative 5S rRNA and is 121 nts long. MT-RNR1 and MT-RNR2 are the two consensus MT rRNAs and are 954 and 1,559 nts long, respectively.

### Defining the “rRNA space”

We define “rRNA space” as the union of (a) the genomic regions that comprise the six rRNAs (see previous paragraph), (b) all rRNA repeats that are listed in RepeatMasker [59] including partial instances, and (c) any *additional* genomic regions that are identified via a *glsearch* [60] search of the genome using the six rRNAs as queries, default parameters, and an *E* value cutoff of 1E−08. An rRF that can be found in the union of the genomic regions obtained through steps a, b, and c above as well as elsewhere in the genome is referred to as an “ambiguous” rRF. Otherwise, it is referred to as being “exclusive” to the rRNA space. This is analogous to our definition of tRNA space and our analyses of tRFs [26, 30, 36, 48].

### Mapping

We first processed the 434 short RNA-seq datasets using *cutadapt* [61] to quality-trim and remove adapters from the sequenced reads. The reads were then mapped to the genome using a brute-force, deterministic, and *exhaustive* approach that enforced exact matching to the genome. Only reads with a minimum of 16 nts were kept and analyzed further. During mapping, we catalogued reads which are exclusive to the rRNA space and which are ambiguous. We also kept track of reads that straddle either the left or the right boundary of any of the six rRNAs (Fig. 1a blue box).

### Thresholding

We thresholded the rRFs using the Threshold-seq tool [35] and default parameter settings. Threshold-seq calculates an adaptive sequence read cutoff that is different for each sample (Fig. 1a green box). We also calculated a ≥ 10 RPM threshold by first normalizing each rRF’s abundance to reads-per-million (RPM) by dividing the number of reads that support the rRF by the total number of sequenced short RNA reads (i.e., read depth) and multiplying by 1 million then keeping only unique rRFs that passed a threshold of ≥ 10 RPM. (Fig. 1a orange box).

### Determining length cutoffs

As might be expected, shorter sequences are more likely than longer sequences to have many genomic instances that are not part of the rRNA space. In fact, we find that many of the identified rRFs with lengths ≥ 16 nts are ambiguous. Thus, for each rRNA in turn, we identified the minimum length at which fewer than 2% of the genomic instances of an rRNA’s rRFs fall outside of the rRNA space. To do this, we first examined rRFs from the same rRNA if and only if their sequence lengths ranged from 16 through 33 nts inclusive. Next, for each rRF, we counted the number of its instances that fall inside the rRNA space, outside the rRNA space, and across the whole genome. For all rRFs from a given rRNA, and for each sequence length value (16–33 nts), we calculated the ratio of the number of instances that fall inside of the rRNA space over the total number of rRFs that fall outside of the rRNA space and call this the signal to noise ratio (S/N). We identified the minimum rRF length for which the S/N becomes ≥ 50 (the number of instances that fall outside of the rRNA space over the total number of genomic instances is ≤ 2%). We repeated this calculation separately for each of the six rRNAs (Fig. 1a red box).

### Analysis

Differential abundances were calculated using the Significance Analysis of Microarrays (SAM) package in R using a stringent false discovery rate (FDR) cutoff of 0.01. Partial least squares-discriminant analysis (PLS-DA) was carried out in R using the default settings and a VIP cutoff of 1.5. Pearson correlations were calculated using R.

### RNA isolation

For total RNA preparation, cells were grown in suspension using RPMI 1640 media with 30% non-heat inactivated FBS + glutamate (Sigma-Aldrich). After seeding, cells were grown for 3–5 days and harvested. RNA was isolated using TRIzol extraction (Invitrogen).

### Northern blotting

We purchased commercially available lymphoblastoid cell lines (Coriell Institute) derived from 18 total people from the CEU, GBR, and YRI populations. For each population, we purchased three male samples and three female samples. The cell lines are the following: CEU females (GM12769, GM12807, GM12837) CEU males (GM12884, GM12905, GM12919), YRI females (GM18487, GM18523, GM18870), YRI males (GM18907, GM19203, GM19239), GBR females (HG00122, HG00134, HG00137), and GBR males (HG00243, HG00264, HG01789). As per Coriell’s policy, all cell lines were tested and found to be mycoplasma-free. 5μg of RNA from each cell line was run on a 15% acrylamide/8 M urea gel at 250 V for 45 min. 100 nmol of RNA target cDNA (5.8S 24-mer, GGGCTACGCCTGTCTGAGCGTCGC; 5.8S 21-mer, TACGCCTGTCTGAGCGTCGCT; 5S 18-mer, ACCGGGTGCTGTAGGCTT; 28S 20-mer, CGCGACCTCAGATCAGACGT) served as positive control. Gel was transferred to Hybond™-N^+^ membrane (Amersham Biosciences, catalog number: RPN303B) and transferred at 400 mA for 10 min. Membrane was dried and then cross-linked twice at 120,000 μJ/cm^2^. All membranes were cut so that the top portion could be probed with the 5S rRNA probe (ACGTCTGATCTGAGGTCGCGT)—the loading control, and the bottom portion was probed with the 5.8S 24-mer (GCGACGCTCAGACAGGCGTAGCCC), 5.8S 21-mer (AGCGACGCTCAGACAGGCGTA), 5S 18-mer (ACCGGGTGCTGTAGGCTT), and 28S 20-mer (ACGTCTGATCTGAGGTCGCG). Membranes were pre-hybridized in hybridization buffer (PerfectHyb™ Plus Hybridization Buffer: H7033-1 L) for 30 min rotating at 37 °C. Northern probes were made using the DIG labeling kit (DIG Oligonucleotide 3′-End Labeling Kit, 2nd Gen: 3353575910). For Fig. 5, the 9 LCL female and 9 LCL male RNAs were run on two different gels and the top portions of each membranes were incubated with 2.5 μl of 5S probe and the lower portions of the membranes were incubated with 5 μl of 5.8S 24-mer probe for 16 h rotating at 37 °C. For Additional file 6: Figure S5, female CEU (GM12769), GBR (HG0112), and YRI (GBM18523) RNA was used and uncut membranes were incubated with 5 μl of the corresponding probes. Detection of membranes was done using the DIG detection kit (DIG Wash and Block Buffer Set: 11585762001, Anti-Digoxigenin-AP, Fab fragments: 11093274910, CDP-Star Chemiluminescent Substrate: C0712-100ML) following the manufacturer’s instructions.

### Secondary structures

Secondary structures were generated using the Vienna RNAFold Web Server http://rna.tbi.univie.ac.at/cgi-bin/RNAWebSuite/RNAfold.cgi [62] with default settings. The sequences for which we predicted secondary structures are listed in the “Reference rRNAs” section.

### Deep sequencing of independently obtained commercial cell lines

We purchased commercially available lymphoblastoid cell lines (Coriell Institute) derived from two individuals: one who was a 63-year-old African American male (ND02672) and one who was a 66-year-old Caucasian American male (ND07114). As per Coriell’s policy, all cell lines were tested and found to be mycoplasma-free. Two different libraries were created for each sample: Illumina’s TruSeq Small RNA Library Prep Kit Set (#RS-200) and NEB’s NEBNext Small RNA Prep Set for Illumina (#E7330) at the Jefferson Genomics Core Facility according to the standard kit protocols, which size select for small RNAs. The Illumina NextSeq 3′-adapter is TGGAATTCTCGGGTGCCAAGG, and the NEBNext 3′-adapter is AGATCGGAAGAGCACACGTCT. The samples were all sequenced using the Illumina NextSeq 500 sequencing platform at 75 cycles and 30 million reads.

## Supplementary information

**Additional file 1: Figure S1.** rRFs are also differentially abundant by sex. **S1A**. SAM identified the differentially abundant rRFs shown in yellow, at an FDR threshold of 0.01. PLS-DA identified rRFs with VIP score ≥ 1.5, shown in green. The intersection of the two circles show how many rRFs were identified by both methods. For each rRNA, we calculated a Jaccard index for the rRFs found by the two methods. **S1B.** The Jaccard index of the rRFs from each rRNA is plotted against the median RPM of the iDARs. **S1C.** The table shows the number of iDARs for each population, and separately for males and females.

**Additional file 2: Figure S2.** rRF pileup along the full-length transcript. This figure shows which part of the 5.8S rRNA the rRFs are produced from. The heatmap is scaled by row (sample). The dark magenta indicates high relative abundance while the dark green indicates low relative abundance. Border arrows indicate the boundaries of the 5.8S rRNA transcript of 157 nts. The leftmost arrow points to where rRFs land outside of the canonical rRNA boundary. The arrows pointing to positions 132 and 156 show where within the full-length rRNA the 5.8S 24-mer rRF is located. The rows are grouped by population.

**Additional file 3: Figure S3.** Specific rRFs are also differentially abundant by sex at varying levels. **S3A-B**. Boxplots show the differential abundance of the 5.8S 19-mer UAAUGUGAAUUGCAGGACA and the 5.8S 21-mer UAAUGUGAAUUGCAGGACACA (underlined region is common to both i-rRFs) between males (grey, *n*=207) and females (orange, *n*=227). **S3A.** The 19-mer has a *p*-value of 0.03, Welch’s *t*-test. **S3B.** The 21-mer has a *p*-value of 0.007, Welch’s t-test.

**Additional file 4.** : Excel sheet containing supporting data showing: 1) the 16,279 rRF sequences that pass Threshold-seq, 10 RPM, and Length Cutoff thresholds, 2) the normalized abundance (RPM) of the i-, 3´-, and x-rRFs from the 3´-end of 5S, and 3) the 5´-rRFs from the 28S rRFs, for the 293T cells and 293T derived EV.

**Additional file 5: Figure S4.** Persistence of rRF profiles across laboratories and different library preparation methods. **S4A**. Pairwise Pearson correlations of the top 1,000 rRFs across 35 samples (five LCL samples sequenced at seven sequencing centers). Color bar labeling: CEU—purple; FIN—orange; GBR—cyan; TSI—gray; and YRI—yellow. **S4B.** Pairwise Pearson correlations of the top 1,000 rRFs across two commercially-available LCLs that we sequenced using two different cDNA library preparation kits (Illumina’s TruSeq and NEB’s NEBNext kits). Color bar labeling: 63 years old African American male (ND02672) is green and 66 years old Caucasian American male (ND07114) is purple.

**Additional file 6: Figure S5.** Presence of rRFs in the context of parental rRNAs and structures S5A-D (top). Northern blots probing for the 24-mer 5.8S i-rRF (GGGCUACGCCUGUCUGAGCGUCGC), 21-mer 5.8S i-rRF (UACGCCUGUCUGAGCGUCGCU), 18-mer 5S i-rRF (ACCGGGUGCUGUAGGCUU), and 20-mer 28S 5´-rRF (CGCGACCUCAGAUCAGACGU) in three female LCLs: CEU (GM12769), GBR (HG0112), and YRI (GBM18523). 5 μg of RNA was used along with 5 pmol of 5.8S or target cDNA and uncut membranes were labeled with 5 μl of the corresponding probes. **S5A-D (bottom).** Predicted secondary structures with minimum free energy scores for each rRF.

**Additional file 7: Figure S6.** rRF sequences aligned to the secondary structure of the full-length rRNA. **S6A-C**. Blue lines highlight the location of the 24-mer 5.8S i-rRF (GGGCUACGCCUGUCUGAGCGUCGC), 21-mer 5.8S i-rRF (UACGCCUGUCUGAGCGUCGCU), 18-mer 5S i-rRF (ACCGGGUGCUGUAGGCUU), and 20-mer 28S 5´-rRF (CGCGACCUCAGAUCAGACGU) on the predicted secondary structures of 5.8S, 5S, and 28S rRNAs, respectively. Minimum free energy scores are also shown for each rRNA. Red arrows indicate the position within the full-length rRNAs from which the rRFs arise.

## Data Availability

All data generated or analyzed during this study are included in this published article, its supplementary information files, and publicly available repositories. The LCL datasets analyzed supporting the conclusion of this article (and its additional files) are available in the International Genome Sequencing Resource (IGSR), ftp://ftp.1000genomes.ebi.ac.uk/vol1/ftp/ [32]. The three 293T datasets and three 293T derived extracellular vesicle (EV) datasets analyzed supporting the conclusion of this article are available in the NCBI GEO repository, https://www.ncbi.nlm.nih.gov/geo/query/acc.cgi?acc=GSE99430 [34]. Accession numbers for each sample used are in the “Other datasets: Gene Expression Omnibus and The Cancer Genome Atlas” section, and the data values for Fig. 6 are in Additional file 4. The 80 uveal melanoma (UVM) datasets analyzed supporting the conclusion of this article are based upon data generated by the TCGA Research Network: https://www.cancer.gov/tcga [33]. The four independent LCL datasets generated supporting the conclusions of this article (and its additional files) are available in the NCBI SRA repository, https://www.ncbi.nlm.nih.gov/bioproject/PRJNA595596.

## Abbreviations

miRNA: MicroRNA
isomiR: MicroRNA isoform
tRNA: Transfer RNA
tRF: Transfer RNA-derived fragment
rRNA: Ribosomal RNA
rRF: Ribosomal RNA-derived fragment
mRNA: Messenger RNA
ncRNA: Non-coding RNA
LCL: Lymphoblastoid cell line
1KG: 1000 Genomes
LSU: Large ribosomal subunit
SSU: Small ribosomal subunit
MT: Mitochondrion/Mitochondria/Mitochondrial
S/N: “Signal-to-noise” ratio
CEU: Utah Residents with Northern and Western European Ancestry (1000 Genomes Project)
FIN: Finnish in Finland (1000 Genomes Project)
GBR: British from England and Scotland (1000 Genomes Project)
TSI: Toscani in Italia (1000 Genomes Project)
YRI: Yoruba in Ibadan, Nigeria (1000 Genomes Project)
SAM: Significance Analysis of Microarrays
PLS-DA: Partial least squares-discriminant analysis
DA: Differential abundance
iDARs: rRFs belonging to the intersection of differentially abundant rRFs that are identified by both SAM and PLS-DA
NGS: Next generation sequencing
GEO: Gene Expression Omnibus
EV: Extracellular vesicle
UVM: Uveal melanoma
TCGA: The Cancer Genome Atlas
nts: Nucleotides
RPM: Reads-per-million
FDR: False discovery rate
MFE: Minimum free energy
RT: Reverse transcription
5′-rRFs: rRFs whose sequences begin at position 1 of the rRNA transcript
3′-rRFs: rRFs whose sequences end at the last position of the rRNA transcript
i-tRFs: rRFs whose start and end positions are internal to the rRNA transcript
x-rRFs: rRFs whose sequences straddle the 5′ or 3′ terminus of the rRNA transcript
