# Supplementary material for: Ribosomal RNA fragmentation into short RNAs (rRFs) is modulated in a sex- and population of origin-specific manner
Source: BMC Biol. 2020 Apr 13;18:38. doi: 10.1186/s12915-020-0763-0 (PMC7153239; doi:10.1186/s12915-020-0763-0)
Supplement: Supplementary file 1 — Additional file 1: Figure S1. rRFs are also differentially abundant by sex. S1A. SAM identified the differentially abundant rRFs shown in yellow, at an FDR threshold of 0.01. PLS-DA identified rRFs with VIP score ≥ 1.5, shown in green. The intersection of the two circles show how many rRFs were identified by both methods. For each rRNA, we calculated a Jaccard index for the rRFs found by the two methods. S1B. The Jaccard index of the rRFs from each rRNA is plotted against the median RPM of the iDARs. S1C. The table shows the number of iDARs for each population, and separately for males and females. [file 12915_2020_763_MOESM1_ESM.pdf]

Additional File 1: Figure S1

S1A

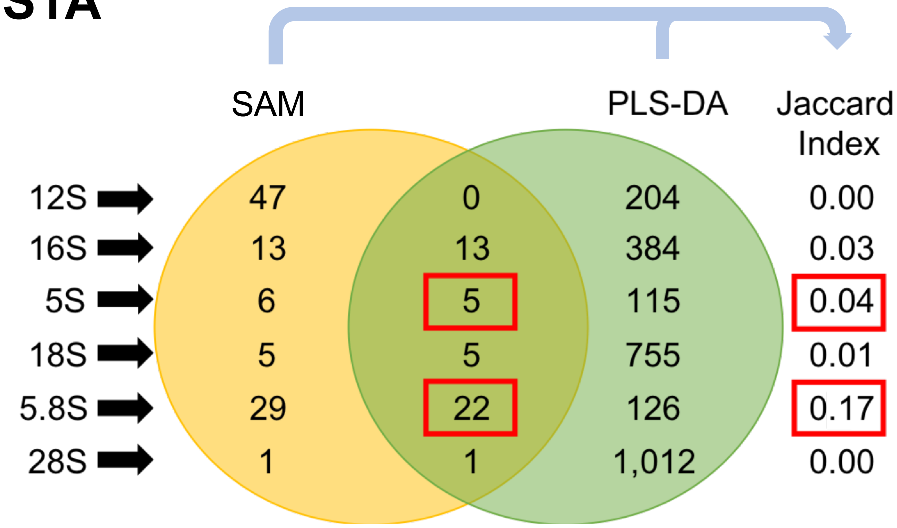

S1B

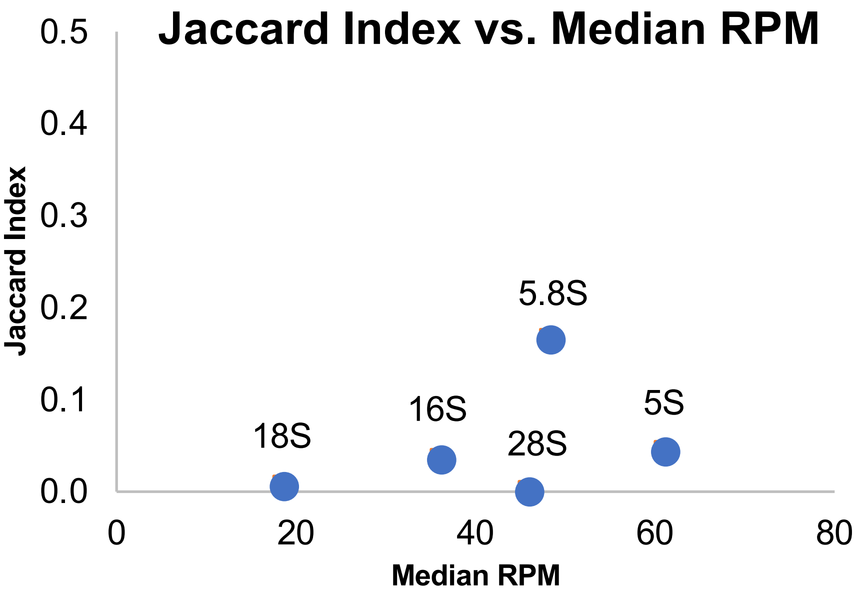

S1C

iDAR by population and rRNA

		12S	16S	5S	18S	5.8S	28S
CEU	males						
	females			2		12	
FIN	males						
	females		1	1	3		
GBR	males						1
	females		2			6	
TSI	males		2				
	females					4	
YRI	males						
	females		8	2	2		
