# Supplementary figures and images for: Ribosomal RNA fragmentation into short RNAs (rRFs) is modulated in a sex- and population of origin-specific manner
Source: BMC Biol. 2020 Apr 13;18:38. doi: 10.1186/s12915-020-0763-0 (PMC7153239; doi:10.1186/s12915-020-0763-0)

## Additional File 2: Figure S2

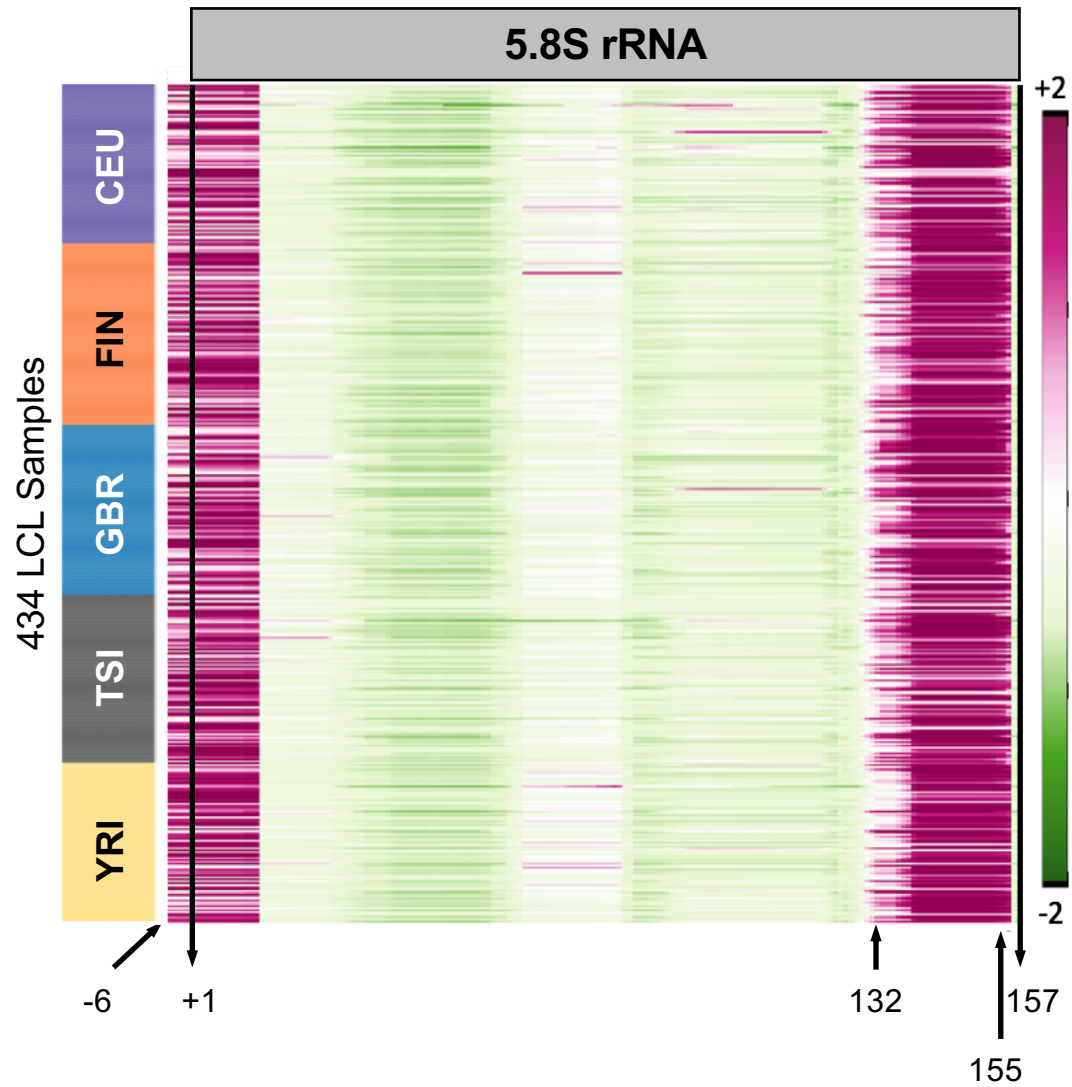

Supplement: Supplementary file 2 — Additional file 2: Figure S2. rRF pileup along the full-length transcript. This figure shows which part of the 5.8S rRNA the rRFs are produced from. The heatmap is scaled by row (sample). The dark magenta indicates high relative abundance while the dark green indicates low relative abundance. Border arrows indicate the boundaries of the 5.8S rRNA transcript of 157 nts. The leftmost arrow points to where rRFs land outside of the canonical rRNA boundary. The arrows pointing to positions 132 and 156 show where within the full-length rRNA the 5.8S 24-mer rRF is located. The rows are grouped by population. [file 12915_2020_763_MOESM2_ESM.pdf]

Additional File 5: Figure S4

S4A

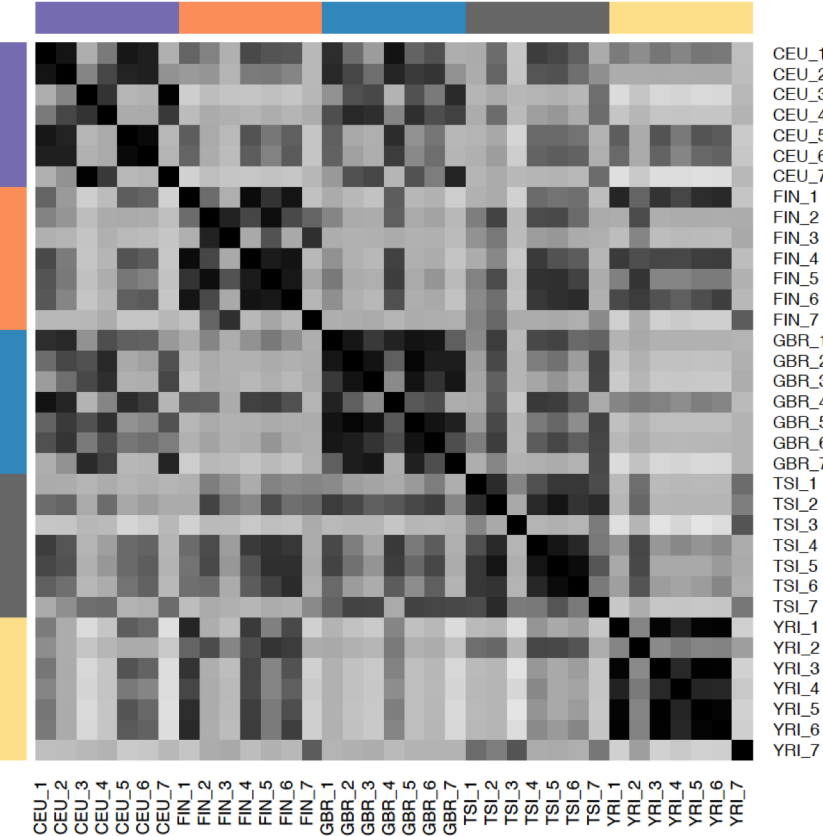

5 LCL Populations

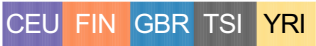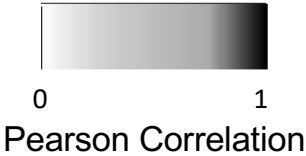

S4B

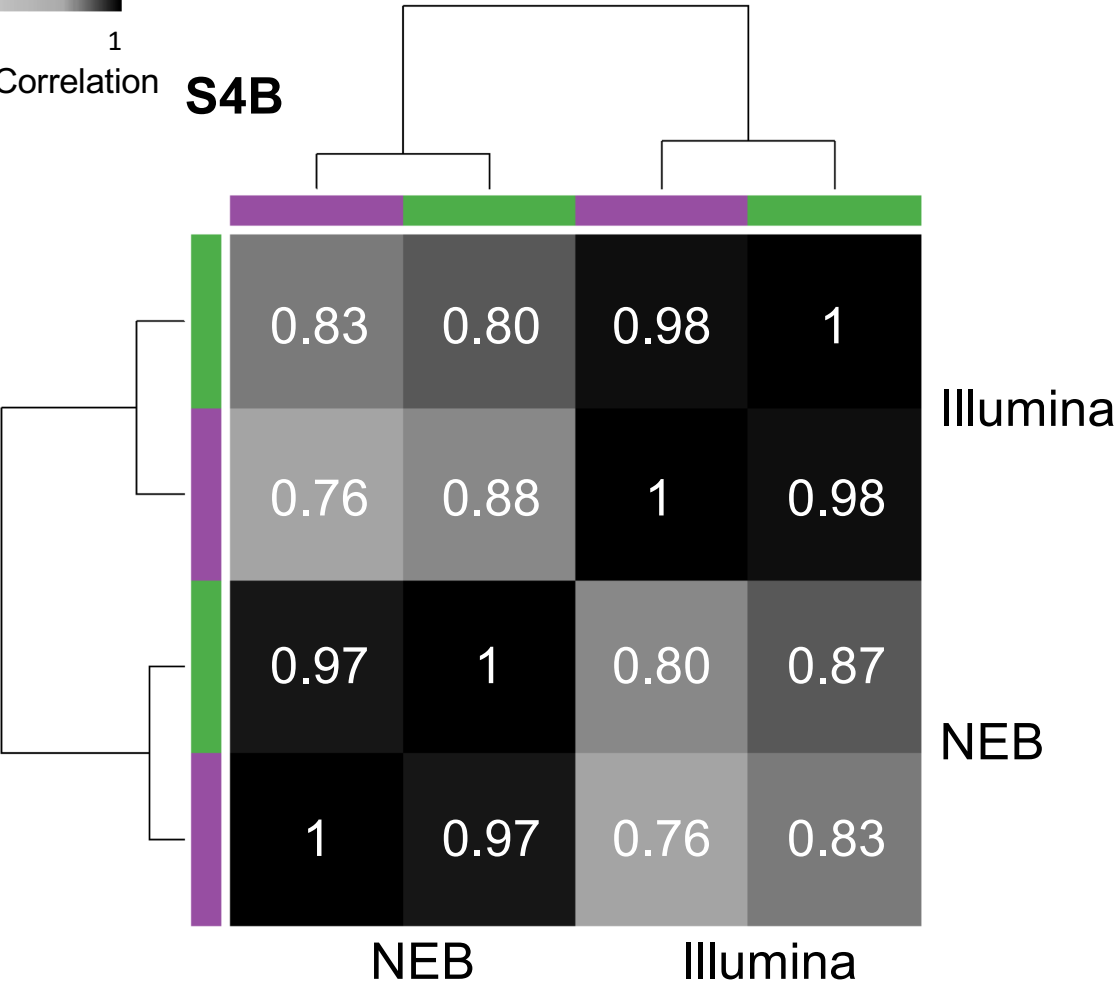

2 Coriel LCLs

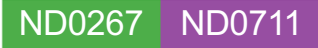

Supplement: Supplementary file 5 — Additional file 5: Figure S4. Persistence of rRF profiles across laboratories and different library preparation methods. S4A. Pairwise Pearson correlations of the top 1,000 rRFs across 35 samples (five LCL samples sequenced at seven sequencing centers). Color bar labeling: CEU—purple; FIN—orange; GBR—cyan; TSI—gray; and YRI—yellow. S4B. Pairwise Pearson correlations of the top 1,000 rRFs across two commercially-available LCLs that we sequenced using two different cDNA library preparation kits (Illumina’s TruSeq and NEB’s NEBNext kits). Color bar labeling: 63 years old African American male (ND02672) is green and 66 years old Caucasian American male (ND07114) is purple. [file 12915_2020_763_MOESM5_ESM.pdf]

Additional File 6: Figure S5

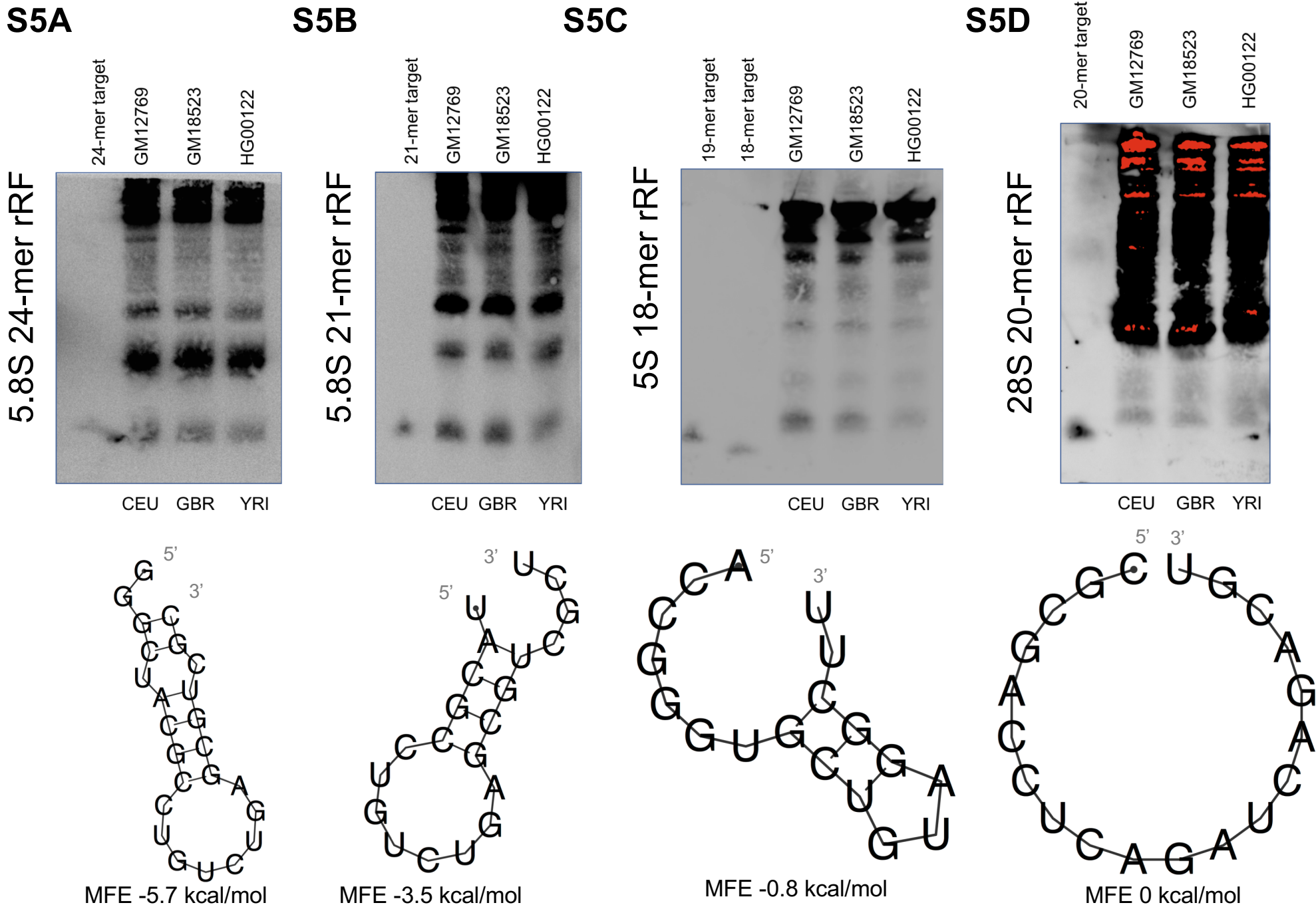

Supplement: Supplementary file 6 — Additional file 6: Figure S5. Presence of rRFs in the context of parental rRNAs and structures S5A-D (top). Northern blots probing for the 24-mer 5.8S i-rRF (GGGCUACGCCUGUCUGAGCGUCGC), 21-mer 5.8S i-rRF (UACGCCUGUCUGAGCGUCGCU), 18-mer 5S i-rRF (ACCGGGUGCUGUAGGCUU), and 20-mer 28S 5´-rRF (CGCGACCUCAGAUCAGACGU) in three female LCLs: CEU (GM12769), GBR (HG0112), and YRI (GBM18523). 5 μg of RNA was used along with 5 pmol of 5.8S or target cDNA and uncut membranes were labeled with 5 μl of the corresponding probes. S5A-D (bottom). Predicted secondary structures with minimum free energy scores for each rRF. [file 12915_2020_763_MOESM6_ESM.pdf]

Additional File 7: Figure S6

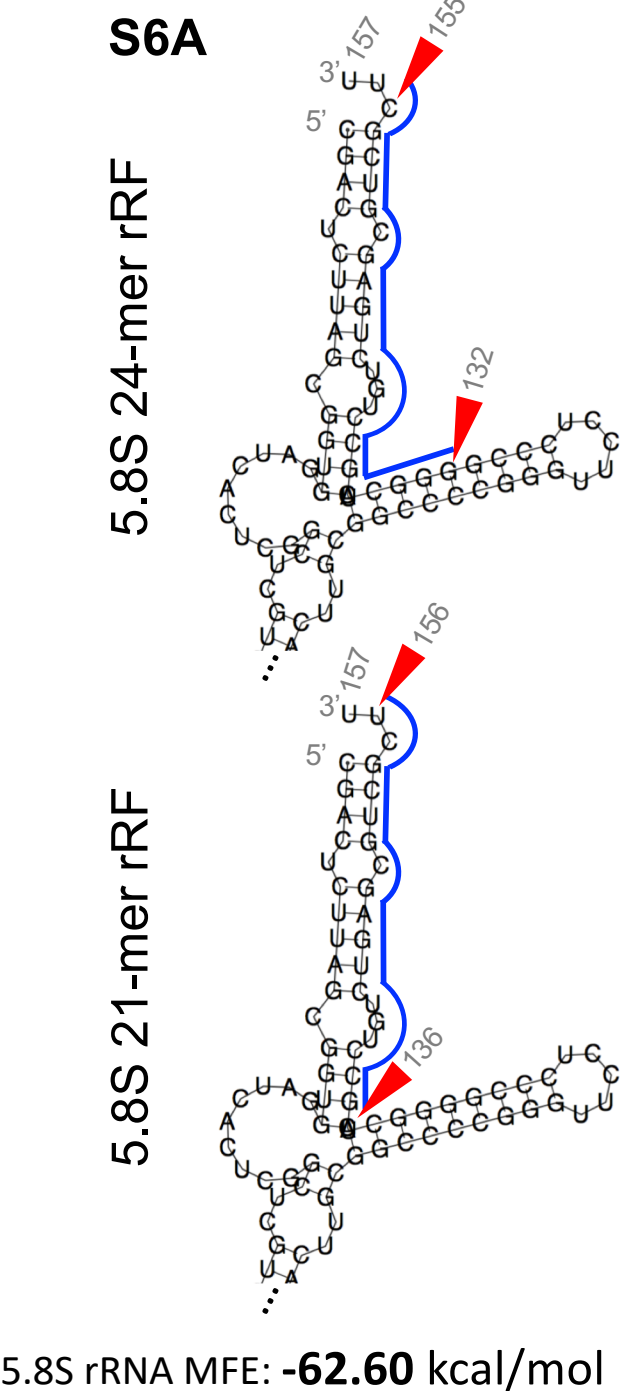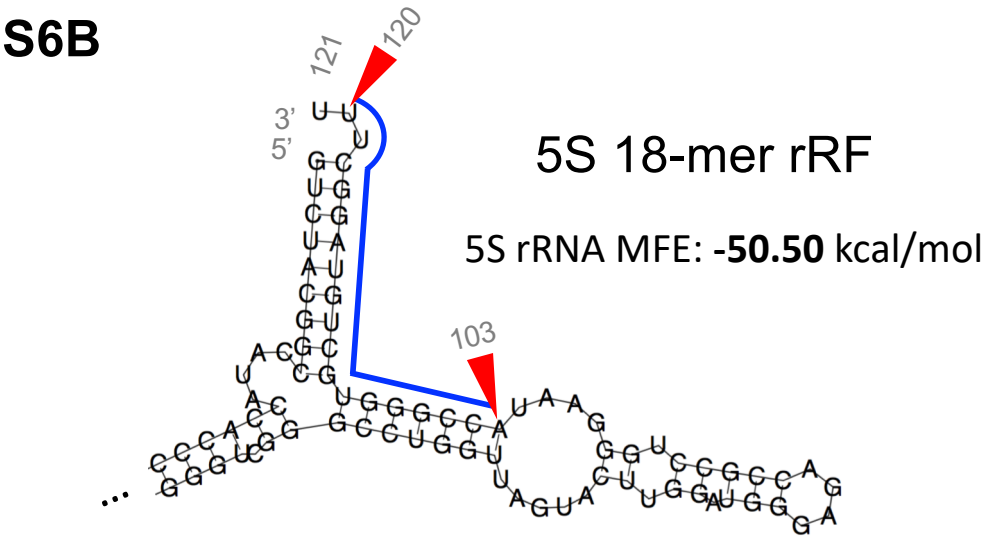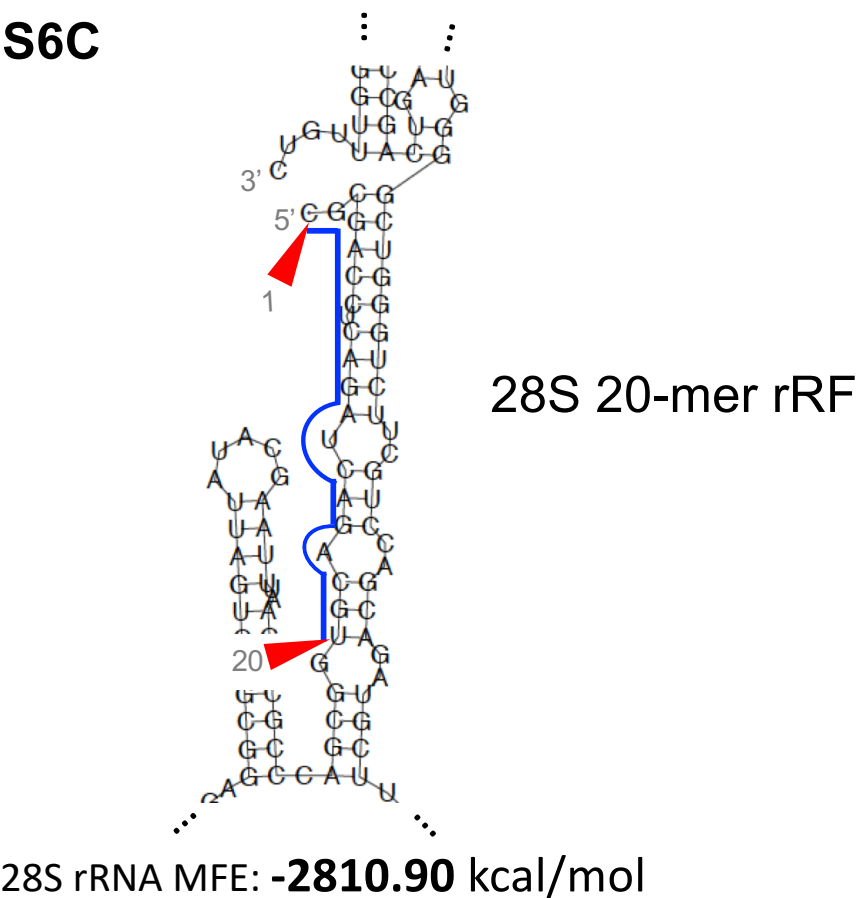

Supplement: Supplementary file 7 — Additional file 7: Figure S6. rRF sequences aligned to the secondary structure of the full-length rRNA. S6A-C. Blue lines highlight the location of the 24-mer 5.8S i-rRF (GGGCUACGCCUGUCUGAGCGUCGC), 21-mer 5.8S i-rRF (UACGCCUGUCUGAGCGUCGCU), 18-mer 5S i-rRF (ACCGGGUGCUGUAGGCUU), and 20-mer 28S 5´-rRF (CGCGACCUCAGAUCAGACGU) on the predicted secondary structures of 5.8S, 5S, and 28S rRNAs, respectively. Minimum free energy scores are also shown for each rRNA. Red arrows indicate the position within the full-length rRNAs from which the rRFs arise. [file 12915_2020_763_MOESM7_ESM.pdf]
