# Supplementary material for: Ribosomal RNA fragmentation into short RNAs (rRFs) is modulated in a sex- and population of origin-specific manner
Source: BMC Biol. 2020 Apr 13;18:38. doi: 10.1186/s12915-020-0763-0 (PMC7153239; doi:10.1186/s12915-020-0763-0)

Additional File 3: Figure S3

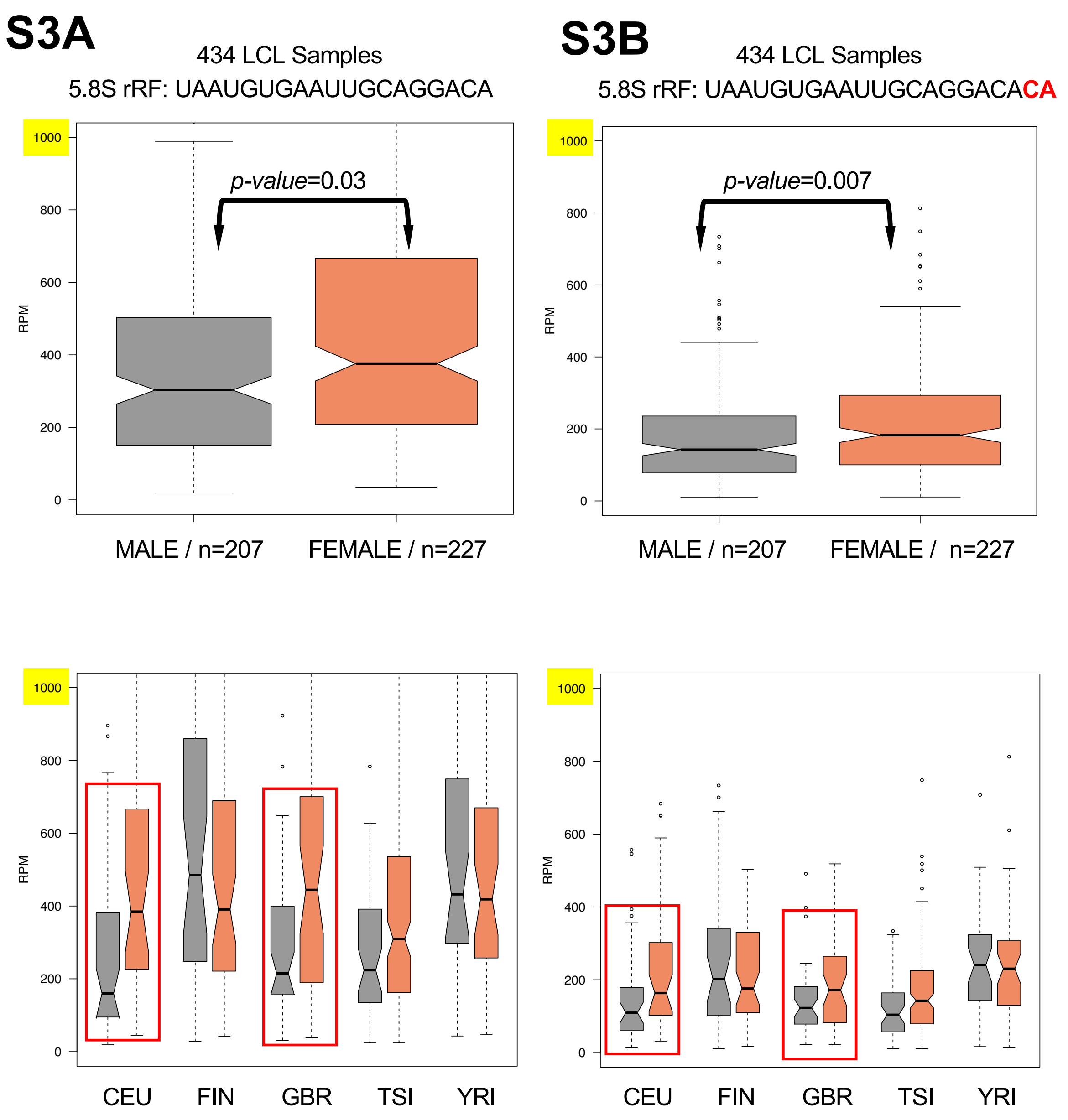

Supplement: Supplementary file 3 — Additional file 3: Figure S3. Specific rRFs are also differentially abundant by sex at varying levels. S3A-B. Boxplots show the differential abundance of the 5.8S 19-mer UAAUGUGAAUUGCAGGACA and the 5.8S 21-mer UAAUGUGAAUUGCAGGACACA (underlined region is common to both i-rRFs) between males (grey, n=207) and females (orange, n=227). S3A. The 19-mer has a p-value of 0.03, Welch’s t-test. S3B. The 21-mer has a p-value of 0.007, Welch’s t-test. [file 12915_2020_763_MOESM3_ESM.pdf]
